# Comparative whole genome analysis reveals re-emergence of human Wa-like and DS-1-like G3 rotaviruses after Rotarix vaccine introduction in Malawi

**DOI:** 10.1093/ve/vead030

**Published:** 2023-05-22

**Authors:** Chimwemwe Mhango, Akuzike Banda, End Chinyama, Jonathan J Mandolo, Orpha Kumwenda, Chikondi Malamba-Banda, Kayla G Barnes, Benjamin Kumwenda, Kondwani C Jambo, Celeste M Donato, Mathew D Esona, Peter N Mwangi, A Duncan Steele, Miren Iturriza-Gomara, Nigel A Cunliffe, Valentine N Ndze, Arox W Kamng’ona, Francis E Dennis, Martin M Nyaga, Chrispin Chaguza, Khuzwayo C Jere

**Affiliations:** Malawi-Liverpool-Wellcome Clinical Research Programme, Kamuzu University of Health Sciences, Blantyre 312225, Malawi; Department of Biomedical Sciences, School of Life Sciences and Allied Health Professions, Kamuzu University of Health Sciences, Blantyre 312225, Malawi; Department of Computer Science, Faculty of Science, University of Malawi, Zomba 305205, Malawi; Malawi-Liverpool-Wellcome Clinical Research Programme, Kamuzu University of Health Sciences, Blantyre 312225, Malawi; Malawi-Liverpool-Wellcome Clinical Research Programme, Kamuzu University of Health Sciences, Blantyre 312225, Malawi; Department of Biomedical Sciences, School of Life Sciences and Allied Health Professions, Kamuzu University of Health Sciences, Blantyre 312225, Malawi; Malawi-Liverpool-Wellcome Clinical Research Programme, Kamuzu University of Health Sciences, Blantyre 312225, Malawi; Malawi-Liverpool-Wellcome Clinical Research Programme, Kamuzu University of Health Sciences, Blantyre 312225, Malawi; Institute of Infection, Veterinary and Ecological Sciences, University of Liverpool, Liverpool L69 7BE, UK; Department of Biological Sciences, Academy of Medical Sciences, Malawi University of Science and Technology, Thyolo 310105, Malawi; Department of Medical Laboratory Sciences, Faculty of Biomedical Sciences and Health Profession, Kamuzu University of Health Sciences, Blantyre 312225, Malawi; Malawi-Liverpool-Wellcome Clinical Research Programme, Kamuzu University of Health Sciences, Blantyre 312225, Malawi; Department of Biomedical Sciences, School of Life Sciences and Allied Health Professions, Kamuzu University of Health Sciences, Blantyre 312225, Malawi; Malawi-Liverpool-Wellcome Clinical Research Programme, Kamuzu University of Health Sciences, Blantyre 312225, Malawi; Department of Clinical Sciences, Liverpool School of Tropical Medicine, Liverpool L3 5QA, UK; Enteric Diseases Group, Murdoch Children’s Research Institute, 50 Flemington Road, Parkville, Melbourne 3052, Australia; Department of Paediatrics, The University of Melbourne, Parkville, Victoria 3010, Australia; Diarrhoeal Pathogens Research Unit, Sefako Makgatho Health Sciences University, Medunsa, Pretoria 0204, South Africa; Next Generation Sequencing Unit and Division of Virology, Faculty of Health Sciences, University of Free State, Bloemfontein 9300, South Africa; Diarrhoeal Pathogens Research Unit, Sefako Makgatho Health Sciences University, Medunsa, Pretoria 0204, South Africa; Centre for Vaccine Innovation and Access, Program for Appropriate Technology in Health (PATH), Geneva 1218, Switzerland; Institute of Infection, Veterinary and Ecological Sciences, University of Liverpool, Liverpool L69 7BE, UK; NIHR Health Protection Research Unit in Gastrointestinal Infections, University of Liverpool, Liverpool L69 7BE, UK; Faculty of Health Sciences, University of Buea, PO Box 63, Buea, Cameroon; Malawi-Liverpool-Wellcome Clinical Research Programme, Kamuzu University of Health Sciences, Blantyre 312225, Malawi; Department of Biomedical Sciences, School of Life Sciences and Allied Health Professions, Kamuzu University of Health Sciences, Blantyre 312225, Malawi; Department of Electron Microscopy and Histopathology, Noguchi Memorial Institute for Medical Research, University of Ghana, Accra, P. O. Box LG 581, Legon, Ghana; Institute of Infection, Veterinary and Ecological Sciences, University of Liverpool, Liverpool L69 7BE, UK; Department of Epidemiology of Microbial Diseases, Yale School of Public Health, Yale University, New Haven, Connecticut 06510, USA; NIHR Mucosal Pathogens Research Unit, Division of Infection and Immunity, University College London, London WC1E 6BT, UK; Yale Institute for Global Health, Yale University, New Haven, Connecticut 06510, USA; Malawi-Liverpool-Wellcome Clinical Research Programme, Kamuzu University of Health Sciences, Blantyre 312225, Malawi; Institute of Infection, Veterinary and Ecological Sciences, University of Liverpool, Liverpool L69 7BE, UK; Department of Medical Laboratory Sciences, Faculty of Biomedical Sciences and Health Profession, Kamuzu University of Health Sciences, Blantyre 312225, Malawi; NIHR Health Protection Research Unit in Gastrointestinal Infections, University of Liverpool, Liverpool L69 7BE, UK; Next Generation Sequencing Unit and Division of Virology, Faculty of Health Sciences, University of Free State, Bloemfontein 9300, South Africa

**Keywords:** rotavirus, G3 strains, whole genome sequencing, sub-Saharan Africa, Malawi, phylodynamic, reassortment, importation, genotype re-emergence, Rotarix vaccine

## Abstract

G3 rotaviruses rank among the most common rotavirus strains worldwide in humans and animals. However, despite a robust long-term rotavirus surveillance system from 1997 at Queen Elizabeth Central Hospital in Blantyre, Malawi, these strains were only detected from 1997 to 1999 and then disappeared and re-emerged in 2017, 5 years after the introduction of the Rotarix rotavirus vaccine. Here, we analysed representative twenty-seven whole genome sequences (G3P[4], *n *= 20; G3P[6], *n *= 1; and G3P[8], *n *= 6) randomly selected each month between November 2017 and August 2019 to understand how G3 strains re-emerged in Malawi. We found four genotype constellations that were associated with the emergent G3 strains and co-circulated in Malawi post-Rotarix vaccine introduction: G3P[4] and G3P[6] strains with the DS-1-like genetic backbone genes (G3-P[4]-I2-R2-C2-M2-A2-N2-T2-E2-H2 and G3-P[6]-I2-R2-C2-M2-A2-N2-T2-E2-H2), G3P[8] strains with the Wa-like genetic backbone genes (G3-P[8]-I1-R1-C1-M1-A1-N1-T1-E1-H1), and reassortant G3P[4] strains consisting of the DS-1-like genetic backbone genes and a Wa-like NSP2 (N1) gene (G3-P[4]-I2-R2-C2-M2-A2-N1-T2-E2-H2). Time-resolved phylogenetic trees demonstrated that the most recent common ancestor for each ribonucleic acid (RNA) segment of the emergent G3 strains was between 1996 and 2012, possibly through introductions from outside the country due to the limited genetic similarity with G3 strains which circulated before their disappearance in the late 1990s. Further genomic analysis revealed that the reassortant DS-1-like G3P[4] strains acquired a Wa-like NSP2 genome segment (N1 genotype) through intergenogroup reassortment; an artiodactyl-like VP3 through intergenogroup interspecies reassortment; and VP6, NSP1, and NSP4 segments through intragenogroup reassortment likely before importation into Malawi. Additionally, the emergent G3 strains contain amino acid substitutions within the antigenic regions of the VP4 proteins which could potentially impact the binding of rotavirus vaccine–induced antibodies. Altogether, our findings show that multiple strains with either Wa-like or DS-1-like genotype constellations have driven the re-emergence of G3 strains. The findings also highlight the role of human mobility and genome reassortment events in the cross-border dissemination and evolution of rotavirus strains in Malawi necessitating the need for long-term genomic surveillance of rotavirus in high disease–burden settings to inform disease prevention and control.

## Introduction

Childhood vaccination remains the most effective public health intervention against rotavirus gastroenteritis (Chandran et al. 2010). Despite the introduction of rotavirus vaccines in 114 countries globally (‘VIEW-Hub by IVAC’ n.d.), rotavirus remains the leading etiological agent of acute gastroenteritis in children (Clark et al. 2017) and is associated with approximately 128,500 deaths per annum among children <5 years old worldwide (Troeger et al. 2018). To reduce the global burden of rotavirus gastroenteritis in children, the World Health Organization (WHO) has pre-qualified the use of four rotavirus vaccines: Rotarix (GlaxoSmithKline (GSK), Rixensart, Belgium), RotaTeq (Merck and Co., Whitehouse Station, NJ, USA), ROTAVAC (Bharat Biotech, India), and ROTASIIL (Serum Institute of India Pvt Ltd, India) (Kirkwood and Duncan Steele 2018). These vaccines are highly effective at preventing rotavirus-associated deaths, hospitalisations, and severe gastroenteritis episodes in high-income settings, although lower effectiveness has been reported in low- and middle-income countries (Saha et al. 2021; Cates, Tate, and Parashar 2022; Henschke et al. 2022). The Rotarix vaccine was introduced into Malawi’s Extended Programme of Immunisation in October 2012, and by 2016, over 99 per cent of vaccine coverage was reached (Bennett et al. 2021). The Rotarix vaccine has demonstrated relatively lower effectiveness in Malawi (31.7–70.6 per cent) during the programmatic use compared with that observed in high-income settings, although the effectiveness against severe rotavirus gastroenteritis in the first year of life is higher against homotypic (70 per cent) compared with heterotypic (40–60 per cent) strains (Bar-Zeev et al. 2016).

Rotavirus has a double-stranded RNA (dsRNA) genome and belongs to the *Reoviridae* family. Its genome is housed in eleven RNA segments that encode six structural proteins (VP1–VP4, VP6, and VP7) and up to six non-structural proteins (NSP1–NSP5/6) (Estes et al. 2007). To fully characterise rotavirus strains, a whole genome classification system was devised to assign genotypes to each genome segment (Matthijnssens et al. 2008b). To date, 41 G, 57 P, 31 I, 27 R, 23 C, 23 M, 38 A, 27 N, 27 T, 31 E, and 27 H genotypes have been assigned for the VP7, VP4, VP6, VP1, VP2, VP3, NSP1, NSP2, NSP3, NSP4, and NSP5 genome segments, respectively (https://rega.kuleuven.be/cev/viralmetagenomics/virus-classification). Normally, the inner capsid and non-structural protein genes of human rotaviruses have either a Wa-like (I1-R1-C1-M1-A1-N1-T1-E1-H1), DS-1-like (I2-R2-C2-M2-A2-N2-T2-E2-H2), or AU-1-like (I3-R3-C3-M3-A3-N3-T3-E3-H3) genotype constellation (Matthijnssens and Marc 2012). While most infections are associated with a single genotype, coinfections with rotavirus strains of different genotypes are also common, which provides favourable conditions for generating progeny viruses with reassortant genomic segments. These genomic reassortment events together with high mutation rates arising from an error-prone RNA polymerase (VP1) that lacks proof-reading mechanisms are the main mechanisms of evolution for rotaviruses (Estes et al. 2007).

Clinically, genotypes G1P[8], G2P[4], G3P[8], G4P[8], G9P[8], and G12P[8] are the most common rotavirus strains associated with diarrhoea in under-five children globally (Dóró et al. 2014). The distribution of these and other rotavirus genotypes varies by geographical location and appears to depend on the rotavirus vaccination status and the vaccine used in a particular region (Donato et al. 2022). Before the introduction of rotavirus vaccines across the continents, G1P[8] strains were the most predominant globally (Bányai et al. 2012). However, G2P[4] strains have been predominant in some countries using Rotarix rotavirus vaccine, while the G12P[8] genotype has been commonly detected in some countries using RotaTeq rotavirus vaccine (Vizzi et al. 2017; Degiuseppe and Stupka 2018; Roczo-Farkas et al. 2018; Carvalho-Costa et al. 2019; Hungerford et al. 2019). The detection rates of G3 genotype and other sporadically circulating rotavirus strains have increased especially in countries using Rotarix vaccine (Roczo-Farkas et al. 2018; Carvalho-Costa et al. 2019; Wahyuni et al. 2021). While G3 strains were not common in the African continent before rotavirus vaccine introduction, these strains have been reported in several African countries post-vaccination (João et al. 2020; Mhango et al. 2020; Mwanga et al. 2020). Our previous work reported the prevalence of rotavirus strains circulating in Blantyre, Malawi, from 1997 to 2019 (Mhango et al. 2020). We showed that genotypes G1, G4, and G8 were frequently detected before Rotarix rotavirus vaccine introduction, whereas G1, G2, G3, and G12 genotypes were more common during the vaccine era (Mhango et al. 2020). Although G3 strains were last detected between 1997 and 1999 before their re-emergence in 2017 after nearly two decades, they became the most predominant rotavirus strains in Blantyre, Malawi, by the end of 2019 following their re-emergence in 2017 (Mhango et al. 2020).

G3 rotavirus strains are commonly associated with a wide range of host species and ‘P’ genotype combinations (Matthijnssens et al. 2006; Martínez-Laso et al. 2009). Similarly, our group previously reported up to four P genotypes that were associated with the G3 rotaviruses that circulated in Blantyre, Malawi, from 2017 (G3P[4], G3P[6], G3P[8], and G3P[10]) (Mhango et al. 2020). While the G3 strains that have emerged or re-emerged in other countries after the introduction of rotavirus vaccines possess equine-like VP7 genome segment and DS-1-like genetic backbone (Arana et al. 2016; Cowley et al. 2016; Dóró et al. 2016; Komoto et al. 2018; Esposito et al. 2019; Luchs et al. 2019), the complete genotype constellation of G3 strains that emerged in Malawi is not known. It was also unknown how genetically related these G3 strains are to the locally circulating rotavirus strains in Malawi. To address these questions, we generated and analysed the whole genome sequences (WGSs) of G3 strains collected through our robust and long-term hospital-based rotavirus surveillance in Blantyre, Malawi, to investigate their genomic epidemiology and evolution in Malawi and broader international context. Our findings show that re-emergence of G3 rotavirus strains was driven by multiple strains possessing either Wa-like or DS-1-like genetic backbone genes, as well as strains possessing DS-1-like genetic backbones genes with a reassortant NSP2 (N1) gene. These strains had the highest genetic similarity with strains from other countries, highlighting the impact of importation events as a mechanism for reseeding the strains in the post-vaccination era following their nearly two decades’ hiatus in Malawi.

## Results

### Sample characterisation and description

**Figure 1.**
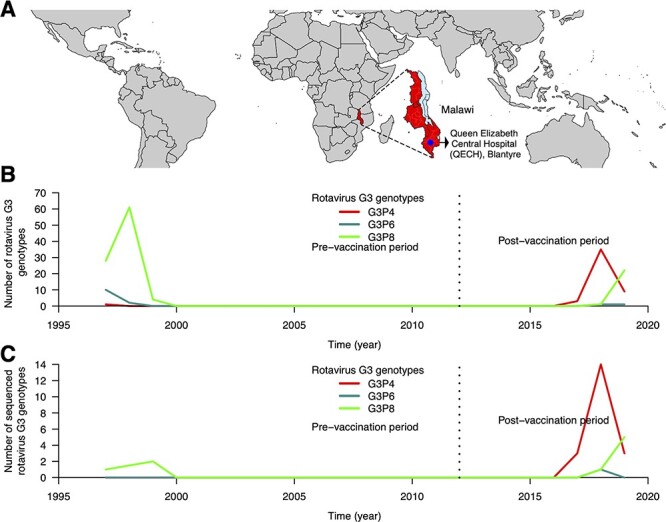
Rotavirus G3 genotypes and WGSs characterised at QECH, Malawi, before and after rotavirus vaccine introduction. (A) Global map highlighting the geographical location of Malawi in Africa as well as the location of QECH in Malawi where the diarrhoea surveillance platform is set up. (B) Absolute number of PCR-positive G3 rotavirus strains genotyped before and after Rotarix rotavirus vaccine introduction at QECH. (C) Absolute number of G3 WGSs generated from stool specimens collected before and after Rotarix rotavirus vaccine introduction in Malawi.

We selected twenty-seven representative G3 strains (20 G3P[4], 1 G3P[6], and 6 G3P[8]) that circulated between November 2017 and August 2019 for WGS analysis (Fig. 1). Overall, the sequence data from our representative strains registered a Phred quality score of *Q *> 30 of which full-length nucleotide sequences of 1,062, 2,359, 1,356, 3,302, 2,684, 2,591, 1,566, 1,059, 1,066, 751, and 664 nucleotides for VP7, VP4, VP6, VP1, VP2, VP3, NSP1, NSP2, NSP3, NSP4, and NSP5 encoding genome segments were generated (Supplementary Table S1). WGSs of three G3P[8] strains detected in 1997 and 1999 that were sequenced from our previous studies were also included in the analysis (Table 1).

**Table 1.** Whole genotype constellations of pre- and post-vaccine Malawian G3 strains. The nomenclature of all the rotavirus strains indicates the rotavirus group, species where the strain was isolated, name of the country where the strain was originally isolated, the common name, year of isolation and the genotypes for genome segment 4 and 9 as proposed by the *Rotavirus Classification Working Group* (RCWG) (Matthijnssens et al. 2008b). Wa-like (genotype 1) gene segments are presented in green while DS-1-like (genotype 2) gene segments are presented in red.

		Genotype constellation											
	Strain nomenclature	VP7	VP4	VP6	VP1	VP2	VP3	NSP1	NSP2	NSP3	NSP4	NSP5	Genogroup/ constellation
	Pre-vaccine strains												
	RVA/Human-wt/MWI/MW1-001/1997/G3P[8]	G3	P[8]	I1	R1	C1	M1	A1	N1	T1	E1	H1	Wa-like
	RVA/Human-wt/MWI/MW1-621/1999/G3P[8]	G3	P[8]	I1	R1	C1	M1	A1	N1	T1	E1	H1	Wa-like
	RVA/Human-wt/MWI/OP1-511/1999/G3P[8]	G3	P[8]	I1	R1	C1	M1	A1	N1	T1	E1	H1	Wa-like
	Post-vaccine strains												
	RVA/Human-wt/MWI/BTY22H/2017/G3P[4]	G3	P[4]	I2	R2	C2	M2	A2	N2	T2	-	H2	DS-1-like
	RVA/Human-wt/MWI/BTY22J/2017/G3P[4]	G3	P[4]	I2	R2	C2	M2	A2	N2	T2	E2	H2	DS-1-like
	RVA/Human-wt/MWI/BTY232/2017/G3P[4]	G3	P[4]	I2	R2	C2	M2	A2	N2	T2	E2	H2	DS-1-like
	RVA/Human-wt/MWI/BTY23I/2018/G3P[4]	G3	P[4]	I2	R2	C2	M2	A2	N2	T2	E2	-	DS-1-like
	RVA/Human-wt/MWI/BTY240/2018/G3P[4]	G3	P[4]	I2	R2	C2	M2	A2	N2	T2	E2	H2	DS-1-like
	RVA/Human-wt/MWI/BTY24R/2018/G3P[4]	G3	P[4]	I2	R2	C2	M2	A2	N2	T2	E2	H2	DS-1-like
	RVA/Human-wt/MWI/BTY25Q/2018/G3P[4]	G3	P[4]	I2	R2	C2	M2	A2	N2	T2	E2	H2	DS-1-like
	RVA/Human-wt/MWI/BTY25L/2018/G3P[4]	G3	P[4]	I2	R2	C2	M2	A2	N2	T2	E2	H2	DS-1-like
	RVA/Human-wt/MWI/BTY26Q/2018/G3P[4]	G3	P[4]	I2	R2	C2	M2	A2	N2	T2	E2	H2	DS-1-like
	RVA/Human-wt/MWI/BTY26H/2018/G3P[4]	G3	P[4]	I2	R2	C2	M2	A2	N2	T2	E2	H2	DS-1-like
	RVA/Human-wt/MWI/BTY27C/2018/G3P[4]	G3	P[4]	I2	R2	C2	M2	A2	N2	T2	E2	H2	DS-1-like
	RVA/Human-wt/MWI/BTY27S/2018/G3P[4]	G3	P[4]	I2	R2	C2	M2	A2	N2	T2	E2	H2	DS-1-like
	RVA/Human-wt/MWI/BTY296/2018/G3P[4]	G3	P[4]	I2	R2	C2	M2	A2	N2	T2	E2	H2	DS-1-like
	RVA/Human-wt/MWI/BTY29D/2018/G3P[4]	G3	P[4]	I2	R2	C2	M2	A2	N2	T2	E2	H2	DS-1-like
	RVA/Human-wt/MWI/BTY29E/2018/G3P[4]	G3	P[4]	I2	R2	C2	M2	A2	N2	T2	E2	-	DS-1-like
	RVA/Human-wt/MWI/BTY2A2/2018/G3P[4]	G3	-	I2	R2	C2	M2	A2	N2	T2	E2	-	DS-1-like
	RVA/Human-wt/MWI/BTY2CM/2018/G3P[6]	G3	P[6]	I2	R2	C2	M2	A2	N2	T2	E2	H2	DS-1-like
	RVA/Human-wt/MWI/BTY2BG/2018/G3P[4]	G3	P[4]	I2	R2	C2	M2	A2	N1	T2	E2	H2	Reassortant
	RVA/Human-wt/MWI/BTY2EP/2019/G3P[4]	G3	P[4]	I2	R2	C2	M2	A2	N1	T2	E2	-	Reassortant
	RVA/Human-wt/MWI/CHX11Q/2019/G3P[4]	G3	P[4]	I2	R2	C2	M2	A2	N1	T2	E2	H2	Reassortant
	RVA/Human-wt/MWI/CHX11X/2019/G3P[4]	G3	P[4]	I2	R2	C2	M2	A2	N1	T2	E2	-	Reassortant
	RVA/Human-wt/MWI/BTY2BD/2018/G3P[8]	G3	P[8]	I1	R1	C1	M1	A1	N1	T1	E1	H1	Wa-like
	RVA/Human-wt/MWI/BTY2EM/2019/G3P[8]	G3	P[8]	I1	R1	C1	M1	A1	N1	T1	E1	H1	Wa-like
	RVA/Human-wt/MWI/BTY2GA/2019/G3P[8]	G3	P[8]	I1	R1	C1	M1	A1	N1	T1	E1	H1	Wa-like
	RVA/Human-wt/MWI/BTY2GC/2019/G3P[8]	G3	P[8]	I1	R1	C1	M1	A1	N1	T1	E1	H1	Wa-like
	RVA/Human-wt/MWI/CHX11U/2019/G3P[8]	G3	P[8]	I1	R1	C1	M1	A1	N1	T1	E1	H1	Wa-like
	RVA/Human-wt/MWI/CHX11S/2019/G3P[8]	G3	P[8]	I1	R1	C1	M1	A1	N1	T1	E1	H1	Wa-like

### Re-emergent G3 rotaviruses in Malawi were associated with four genotype constellations

We analysed WGSs of twenty-seven representative G3 strains to determine the genotype constellations of the viruses that re-emerged in Blantyre, Malawi, during the post-vaccine introduction period. WGS analysis revealed that sixteen G3P[4] (80 per cent, *n = *20) strains had a DS-1-like genotype constellation for the inner capsid and non-structural genome segments (G3P[4]-I2-R2-C2-M2-A2-N2-T2-E2-H2) (Table 1). The only G3P[6] strain that was sequenced also had a DS-1-like genotype constellation (G3P[6]-I2-R2-C2-M2-A2-N2-T2-E2-H2) (Table 1). In contrast, the three G3P[8] strains detected from 1997 to 1999 (33.3 per cent, *n *= 9) before rotavirus vaccine introduction and six from 2018 to 2019 (66.6 per cent, *n *= 9) during the rotavirus vaccine era in Malawi had the Wa-like genotype constellation (G3P[8]-I1-R1-C1-M1-A1-N1-T1-E1-H1) (Table 1). Unexpectedly, four G3P[4] (20 per cent, *n *= 20) strains that were detected towards the end of 2018 had a mosaic genotype constellation consisting of a core DS-1-like backbone genes but with a Wa-like NSP2 gene (N1) in place of the usual N2 gene segment normally associated with the DS-1-like genotype constellation (Table 1). We therefore classified these viruses as reassortant DS-1-like G3P[4] strains. Thus, a total of four genotype G3 variants circulated between 2017 and 2019, namely the typical DS-1-like strains (G3P[4] and G3P[6]), reassortant DS-1-like strains (G3P[4]), and Wa-like strains (G3P[8]), of which the latter two co-circulated from December 2018 till August 2019 (Supplementary Table S2).

We further explored the genomic diversity of the four G3 variants by placing them in the globally used lineage classification systems. We used lineage frameworks by Sadiq et al. (2019) to assess the diversity of the genome segments encoding the outer capsid proteins (VP7 and VP4) and the framework by Agbemabiese et al. (2019) to assess the diversity of the genome segments encoding the inner capsid and non-structural proteins for DS-1-like genotype constellation. We could not do the same for the inner capsid segments of the Wa-like strains since there is no known lineage framework for Wa-like genome segments. WGS analysis revealed a wider genetic diversity (1–4) per genome segment for the emergent four G3 variants (Supplementary Table S3). The highest diversity was observed within VP4, while the lowest was seen in VP7 (Supplementary Table S3). However, we also observed within lineage variation for DS-1-like variants (VP1, VP2, VP3, and NSP3) having up to two, while NSP5 had up to three unique clusters within the same lineage (Supplementary Fig. S3). Although we only identified four variants associated with the G3 rotavirus strains, the detection of multiple lineages within each genome segment suggests a high genetic diversity of the emergent G3 strains that circulated in Blantyre, Malawi, between 2017 and 2019.

### Re-emergent G3 strains were genetically distinct from previous and co-circulating rotaviruses in Malawi

Our present findings revealed a co-circulation of G3 strains with either a Wa-like or a DS-1-like inner capsid and non-structural genome segments late in 2018. We therefore explored the possibility that the reassortant G3P[4] strains acquired a Wa-like NSP2 genome segment through coinfections of Wa-like and DS-1-like strains that co-circulated in Malawi. We constructed a maximum likelihood (ML) phylogenetic tree for all Malawian Wa-like NSP2 (N1) genome segment to explore their genetic relationship to the NSP2 of the reassortant DS-1-like G3P[4] strains. The phylogenetic analysis revealed that the Wa-like NSP2 genome segment (N1 genotype) of the reassortant DS-1-like G3P[4] strains formed a separate monophyletic cluster from the N1 NSP2 genome segments that were associated with co-circulating Wa-like G3P[8] and other locally detected non-G3 strains (Supplementary Fig. S2). The NSP2 genome segment of the reassortant DS-1-like G3P[4] and other co-circulating Wa-like Malawian strains differed by 20–23 single nucleotide polymorphisms (SNPs; 89.82–98.63 per cent nucleotide sequence similarity) within the NSP2 open reading frame (ORF) sequence. These findings suggested that the NSP2 genome segment of the emergent G3 strains was not acquired from strains circulating in Malawi, especially after the re-emergence of the G3, as these could have most likely been detected by our rotavirus surveillance system.

**Figure 2.**
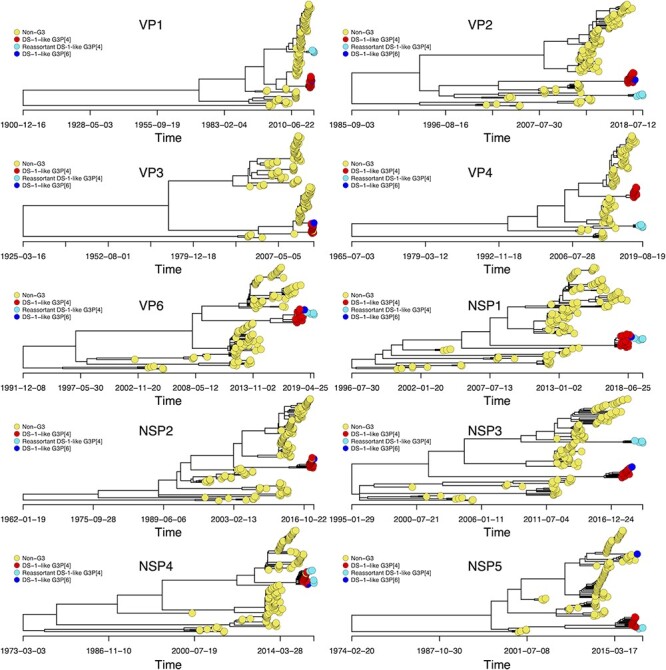
Time-resolved phylogenetic trees for DS-1-like genome segments associated with G3 as well as non-G3 rotavirus strains detected in Malawi from 1997 to 2019. Only previously circulating DS-1-like genome segments with a complete ORF were used to estimate the time to the most common recent ancestor for the DS-1-like (G3P[4], G3P[6], and reassortant G3P[4]) in relation to locally circulating strains. Time trees were constructed using Nextstrain. The VP4 tree only contains genotype P[4].

Previous WGS work in Malawi revealed a circulation of non-G3 genotypes with either a Wa-like or a DS-1-like genomic constellation (Jere et al. 2018). Considering the segmented nature of rotaviruses, we conducted a genome segment–specific time-resolved phylogenetic analysis to assess the genomic similarity of the emergent G3 strains to locally detected co-circulating non-G3 Wa-like or DS-1-like rotaviruses in Malawi. When we employed the time tree analysis to compare the nucleotide sequences of the VP4 and nine backbone genome segments, we observed that the pre- and post-vaccine G3 strains formed separate monophyletic clades from the co-circulating non-G3 Wa-like and DS-1-like strains (Fig. 2 and Supplementary Fig. S1). These findings suggested that the emergent G3 strains did not evolve from or acquire their genome segments from the other co-circulating non-G3 rotaviruses raising the possibility of new introduction of these strains into Malawi.

When we focused on the clustering patterns of the G3 strains, we identified two distinct populations of genome segments encoding both structural and non-structural proteins for Malawian G3P[4] strains that formed separate monophyletic clades. The monophyletic clades for the DS-1-like G3P[4] strains clustered away from the reassortant DS-1-like G3P[4] strains in VP1, VP2, VP4, NSP3, and NSP5 encoding genome segments (Fig. 2). Even though the reassortant DS-1-like G3P[4] strains emerged after the DS-1-like G3P[4] and shared similar NSP1, NSP4, and VP6 genome segments, the time to the most recent common ancestor (tMRCA) of the NSP3, NSP5, VP1, VP2, and VP4 genome segments for the reassortant DS-1-like G3P[4] strains (ranging from 2001 to 2010) (Table 2) and that of the DS-1-like G3P[4] strains (ranging from 2001 to 2008) (Table 2) varied considerably across the respective segments. When we looked at the estimated mutation rates for each of the genome segments (NSP3, NSP5, VP1, VP2, VP3, and VP4) (2.52 × 10^–4^ to 1.67 × 10^–3^ nucleotide substitutions per site per year) (Table 2), the number of SNPs, and the time between the initial emergence of the two G3P[4] variants, we concluded that there was not enough time for these specific genome segments of reassortant DS-1-like G3P[4] to have evolved from the DS-1-like genome segments. Thus, these data suggested that the DS-1-like G3P[4] and reassortant DS-1-like G3P[4] strains did not share an immediate common ancestor and that the reassortant DS-1-like G3P[4] strains were introduced independently already having a reassortant NSP2 genome segment from a source where DS-1-like G3P[4] strains similar to those detected in Blantyre, Malawi, during this period were also circulating.

**Table 2.** Time to the most recent common ancestor (tMRCA) for Malawian Wa-like and DS-1-like G3 strains and estimated mutation rates for Wa-like and DS-1-like time trees. The time to the most common recent ancestors (tMRCA) for G3 strains were estimated in relation to locally circulating human DS-1-like and Wa-like genome segments. The tMRCA and mutation rates were extracted from the time trees generated in Nextstrain for Figure 2 and Supplementary Figure S1.

	Wa-like G3P[8]		DS-1-like G3P[4]		Reassortant DS-1-like G3P[4]
Genome segment (protein)	tMRCA (95% CI)	Mutation rates^c^	tMRCA (95% CI)	Mutation rates	tMRCA (95% CI)
1 (VP1)	21 May 1996 (25 July 1990 to 20 September 1998)	8.15 × 10^−4^	11 February 2008 (21 July 2006 to 10 May 2009)	8.09 × 10^−4^	12 February 2008 (30 December 2005 to 15 February 2009)
2 (VP2)	3 January 2009 (6 August 2007 to 6 May 2010)	9.35 × 10^−4^	18 October 2002 (21 March 2000 to 15 March 2005)	6.43 × 10^−4^	17 October 2002 (24 November 1999 to 3 September 2004)
3 (VP3)^a^	12 July 2000 (8 October 1999 to 11 November 2001)	1.04 × 10^−3^	5 January 2012 (1 April 2010 to 22 May 2012)	1.67 × 10^−3^	to
4 (VP4)	3 December 2008 (14 November 2007 to 29 March 2010)	8.31 × 10^−4^	7 September 2007 (13 October 2003 to 16 November 2010)	9.14 × 10^−4^	28 May 2010 (16 July 2006 to 20 August 2013)
6 (VP6)	26 November 2006 (28 June 2003 to 24 November 2013)	6.37 × 10^−4^	14 December 2008 (20 October 2002 to 18 November 2013)	8.30 × 10^−4^	14 December 2008 (20 October 2002 to 18 November 2013)
5 (NSP1)	15 March 2001 (21 July 2000 to 17 January 2004)	1.55 × 10^−3^	15 July 2007 (10 September 2004 to 17 July 2009)	1.44 × 10^−3^	15 July 2007 (10 September 2004 to 17 July 2009)
7 (NSP2)^b^	–	–	17 January 2003 (8 January 1996 to 28 January 2005)	6.59 × 10^−4^	–
8 (NSP3)	16 October 2005 (10 June 2003 to 31 May 2008)	4.23 × 10^−4^	21 July 2001 (29 August 1997 to 10 May 2006)	7.55 × 10^−4^	11 May 2009 (13 January 2006 to 24 May 2012)
10 (NSP4)	29 March 2003 (16 August 2000 to 12 June 2004)	8.46 × 10^−4^	25 February 2010 (15 July 2005 to 9 January 2012)	1.43 × 10^−3^	25 February 2010 (15 July 2005 to 9 January 2012)
11 (NSP5)	6 January 1995 (19 November 1984 to 24 June 2001)	2.52 × 10^−4^	21 May 2001 (5 February 1998 to 25 January 2005)	6.09 × 10^−4^	21 May 2001 (5 February 1998 to 25 January 2005)

a tMRCA were not estimated for reassortant DS-1-like G3P[4] genome segments because they resembled animal-like genome segments thus we could not estimate the correct tMRCA using the current dataset.

b tMRCA were not estimated for Wa-like NSP2 genome segments due to a lack of a sufficient molecular clock signal to conduct the analysis.

c Mutation rates were defined as the number of substitutions per site per year.

### Emergent G3 strains resembled typical human rotaviruses likely imported into Malawi

**Figure 3.**
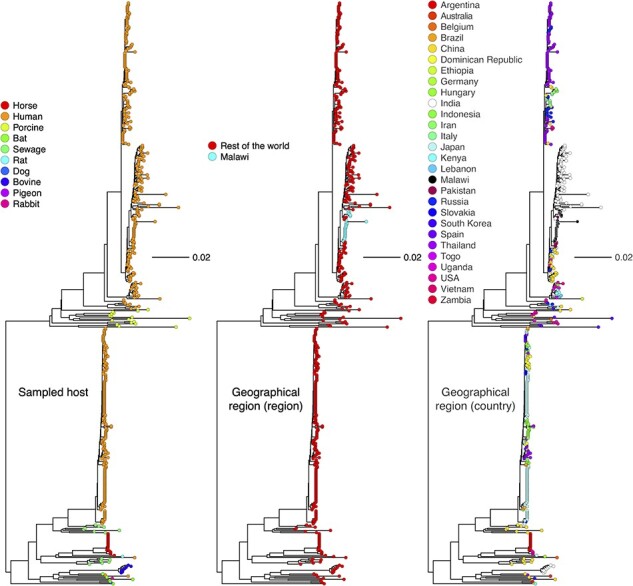
Global ML phylogenetic trees for VP7 genome segments encoding the genotype G3. Only genome segments with a complete ORF characterised between 2010 and 2019 were included in the analysis. The GTR evolutionary model with gamma heterogeneity across nucleotide sites was used for phylogenetic inference while 1,000 bootstraps were used to assess the reliability of the branching order. The tree was rooted using the RVA/Pigeon-wt/JPN/PO-13/1989/G18P[17], but the out-groups were omitted for better visualisation.

**Figure 4.**
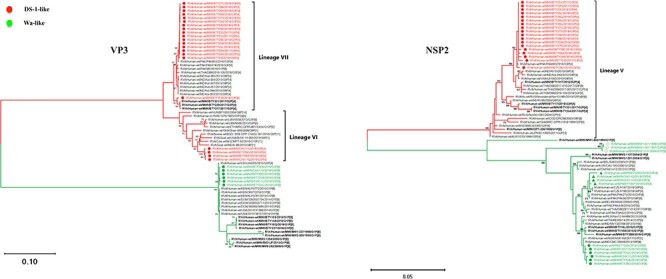
ML phylogenetic trees based on complete ORF of rotavirus VP3 and NSP2 genome segments. The trees were out grouped at RVA/Pigeon-wt/JPN/PO-13/1983/G18P[17] but were omitted for clear visualisation. The GTR evolutionary model with gamma heterogeneity across nucleotide sites was used for phylogenetic inference. Bootstrap values ≥70 per cent are shown adjacent to each branch node. Circles represent post-vaccine, while diamonds represent pre-vaccine Malawian G3 strains. Reassortant G3P[4] strains are represented by triangles in the NSP2 ML tree.

Human G3 rotaviruses have been detected at higher frequencies globally during the past decade with the majority possessing equine-like rotavirus genome segments. While the present Malawi G3 strains had either a DS-1-like or Wa-like genomic constellation, the origins of their genome segments were unclear. To determine the potential host origins of the emergent G3 strains, we performed an ML phylogenetic analysis for the VP7 genome segment of the Malawian and globally detected G3 strains (2010–20). ML analysis revealed that the genome segments encoding VP7 for all Malawian G3 strains clustered together with those of the typical human G3 strains that were detected from various countries across the globe (Fig. 3). Except for the VP3, the rest of the genome segments clustered together with genome segments characterised from other human rotaviruses (Supplementary Fig. S3). The VP3 (M2) genome segments of the reassortant G3P[4] strains clustered together with M2 genome segments commonly characterised in ruminant animals (Fig. 4B). This suggested that intergenogroup reassortment events between human rotaviruses and strains that circulate in Artiodactyla order were part of the evolutionary events that led to the emergence of the reassortant DS-1-like G3P[4] strains.

We then looked at the geographical origin of the genome segments associated with G3 rotaviruses circulating in Malawi. ML analysis revealed that all VP7 genome segments associated with strains possessing a DS-1-like backbone genes (G3P[4], reassortant G3P[4], and G3P[6]) shared a high nucleotide sequence similarity (99.6–99.8 per cent) with only 4 to 6 SNPs observed within the ORF to contemporary G3P[4] strains characterised in the Middle East Asia, Pakistan (Fig. 3 and Supplementary Fig. S3). In contrast, the VP7 genome segment of Wa-like (G3P[8]) strains clustered together with and only had 5 to 7 SNPs within the ORF to contemporary G3P[8] strains characterised in the Far East Asia (Japan and Thailand) (Fig. 3 and Supplementary Fig. S3). The rest of the DS-1-like G3P[4] genome segments clustered together with isolates from the Asian continent with a highest nucleotide sequence similarity (99.40–99.70 per cent) observed against contemporary G3P[4] strains that were detected in Pakistan (Fig. 4 and Supplementary Fig. S3). While the rest of the gene segments for the DS-1-like G3P[6] study strain had a similar clustering pattern to the present study DS-1-like G3P[4] strains, the VP4 gene segment showed a high nucleotide sequence similarity to contemporary P[6] segments characterised in the African continent (99.11–99.53 per cent; Mozambique, Zambia, Zimbabwe, and South Africa) (Supplementary Fig. S3). To the contrary, we observed two clustering patterns of the emergent reassortant DS-1-like G3P[4] strains in Malawi depending on the genome segment. While NSP1, NSP4, and VP6 showed a high nucleotide sequence identity to Asian (Pakistan) isolates, similar to the DS-1-like G3P[4] strains, their NSP2, NSP5, VP1, VP2, and VP4 showed a high nucleotide sequence identity (99.48–99.70 per cent) to cognate genome segments characterised in Europe (Czech Republic) and Asia (Pakistan) (Fig. 4 and Supplementary Fig. S3). On the other hand, the Wa-like G3P[8] gene segments showed similarity to Wa-like segments for non-G3 strains characterised from the African (99.1–99.55 per cent; Zimbabwe, South Africa, Mozambique, Kenya, Nigeria, and Rwanda) and Wa-like G3P[8] from the Asian (99.3–99.73 per cent; Japan, India, and Indonesia) continents (Fig. 4 and Supplementary Fig. S3). Although most of these findings showed a high similarity to rotavirus strains reported from Asian countries, these genome segments are widespread across the African, Asian, European continents and potentially other unsampled settings suggesting a potential for frequent cross-border dissemination.

### Reassortant DS-1-like G3P[4] likely emerged through multiple reassortment events prior to their introduction into Malawi

**Figure 5.**
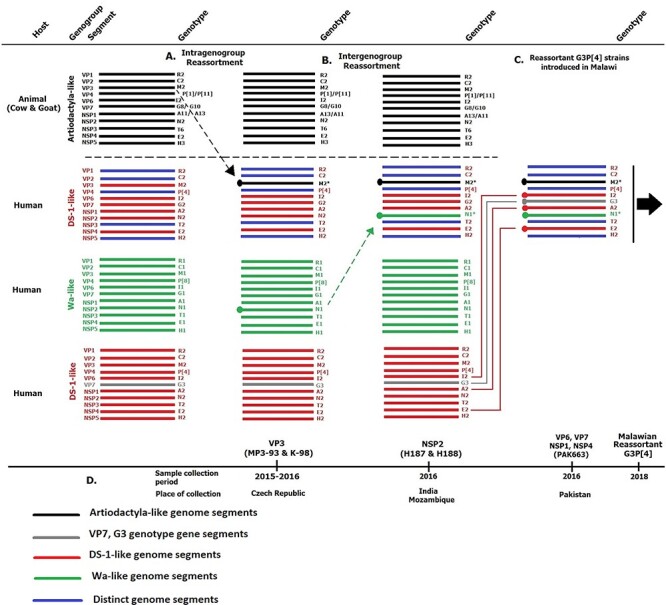
Reconstruction of the sequence of reassortment events that potentially lead to the emergence of the reassortant G3P[4] strains that were detected in Malawi. The reassortment events were hypothesised based on the genetic relationships between the genome segments of the reassortant DS-1-like G3P[4] rotaviruses that were detected in Malawi and those available in the NCBI GenBank to date. (A) The reassortant DS-1-like G3P[4] rotavirus strains that were detected in Malawi likely acquired their VP1, VP2, VP4, NSP1, and NSP5 genome segments from human DS-1-like rotaviruses and their VP3 from artiodactyl rotaviruses that circulated in countries where circulating rotaviruses have not been sequenced yet, or if done, their full-length genome sequences have not yet been deposited to the NCBI GenBank. (B) Both intergenogroup and intragenogroup reassortment events between Wa- and DS-1-like rotaviruses were likely taking place from these unsampled regions, of which various progeny rotavirus populations were possibly generated. Reassortant DS-1-like strains that acquired their NSP2 through intergenogroup reassortment from Wa-like strains, which shared closest nucleotide similarity with rotaviruses that circulated in countries like Czech Republic between 2015 and 2016. (C) Various evolutionary events likely took place between the reassortant DS-1-like, DS-1-like G3P[4], and Wa-like G3P[8] rotaviruses that circulated in regions like Pakistan between 2014 and 2016, and in other unsampled regions that potentially led to various progeny rotavirus populations. One of the resultant progeny populations could be the reassortant DS-1-like G3P[4] strains from (B) that acquired NSP1, NSP4, VP6, and VP7 from the DS-1-like G3P[4] strains that were circulating in regions like Pakistan through complex reassortment events. The resultant reassortant DS-1-like G3P[4] rotaviruses were the ones that were exported to Malawi and caused diarrhoea disease in children that were hospitalised at QECH in Blantyre, Malawi, from 2018 or circulated in unsampled regions. (D) The period and country where the closest related strains to the genome segments of the reassortant DS-1-like G3P[4] were detected. The horizontal lines represent the eleven rotavirus genome segments.

As all genome segments for the reassortant DS-1-like G3P[4] did not cluster with those of the other rotavirus strains that were circulating in Malawi, we used phylogenetic inferences for each genome segment to determine the origin of the reassortant strains. Since the VP1, VP2, VP4, NSP3, and NSP5 encoding genome segments of the reassortant DS-1-like G3P[4] were not closely related to the currently available strains in the GenBank, presumably due to dearth of genomic rotavirus surveillance in many countries, we hypothesised that the reassortant DS-1-like G3P[4] acquired these five genome segments from human rotaviruses that circulated from unsampled locations. Our reassortant strains likely acquired their VP3 genome segment from artiodactyl rotaviruses through intergenogroup reassortment as the closest related cognate VP3 encoding genome segments were those of bovine strain MPT-93 detected in Mozambique in 2015 and a caprine strain K-98 detected in India in 2015 (96.33–96.77 per cent nucleotide sequence similarity). They likely acquired their genome segment encoding NSP2 from Wa-like rotaviruses through intergenogroup reassortment as it was assigned an N1 genotype. Phylogenetically, the genome segment encoding NSP2 of our reassortant strains was closely related (99.48–99.79 per cent nucleotide sequence similarity) to those that were detected in the Czech Republic (RVA/Human-wt/CZE/H186/2018/G9P[4] and RVA/Human-wt/CZE/H187/2018/G9P[4]) with about 2–7 SNP difference within the ORF and Pakistan (RVA/Human-wt/PAK/ PAK274/2015/GXP[8] and RVA/Human-wt/PAK/PAK56/2015/ G9P[8]) with about 2–7 SNP difference within the ORF. However, we could not infer when and where exactly the NSP2 genome reassortment events occurred due to the limited numbers of available rotavirus WGS data from many countries. Similarly, the reassortant DS-1-like G3P[4] likely acquired or donated their VP6, VP7, NSP1, and NSP4 encoding genome segments from or to DS-1-like G3P[4] rotaviruses that circulated in Islamabad and Rawalpindi in Pakistan from 2014 to 2016 (Umair et al. 2018; Sadiq et al. 2019; Naqvi et al. 2020) As limited countries are conducting rotavirus genomic surveillance, it is possible that these reassortment events occurred in an unsampled location prior to their importation into Pakistan. Nevertheless, the resultant reassortant DS-1-like G3P[4] strains were the ones that likely ended up in Malawi where they were associated with diarrhoea infections in 2017 and 2018 at Queen Elizabeth Central Hospital (QECH) (Mhango et al. 2020) (Fig. 5). Thus, the reassortant DS-1-like G3P[4] strains were likely generated through a series of reassortment events elsewhere prior to their circulation in Malawi.

### Amino acid substitutions within antigenic regions of G3P[8] have potential to drive vaccine escape variants

Antigenic regions of the rotavirus outer capsid proteins are critical for inducing neutralising antibodies (Estes et al. 2007). We assessed if there were amino acid differences between antigenic regions of the Malawian G3s strains to that of the G1P[8] Rotarix vaccine strain and determined the structural conformational differences resulting from these mismatches. Due to the known amino acid mismatches between heterotypic rotavirus genotypes (Matthijnssens et al. 2008a), this analysis was limited to homotypic genotypes; hence, only the VP4 proteins of the G3P[8] strains were compared to that of the G1P[8] Rotarix vaccine strain (P[8] genotype). At least 32 out of the 36 amino acids that span across the antigenic regions along the VP5* and VP8* subunits of the VP4 for the G3P[8] strains matched those of the Rotarix strain (Supplementary Table S4). The VP5* subunits of the study G3P[8] strains (Lineage III) were identical to that of Rotarix strain (Lineage I) across all antigenic regions (Supplementary Table S4). However, differences were observed within the VP8*-1 and VP8*-3 antigenic regions. The E150D was the only substitution along the VP8*-1 antigenic region, whereas S125N, S131R, and N135D substitutions occurred within the VP8*-3 antigenic region (Supplementary Table S4). The S131R amino acid substitution within the VP8*-3 resulted in a change from a charged amino acid with a potential of five hydrogen bonds to a polar amino acid with four hydrogen bonds. When we aligned the VP8* protein structures of the G3P[8] study strains against that of Rotarix strain, the superimposed structures revealed structural conformation differences within antigenic epitope region 8*-1 and 8*-3 specifically at positions 150 and 131, respectively (Supplementary Fig. S4). The differences in the structural conformation of the antigenic region have potential to impact the neutralising ability of the Rotarix vaccine–induced antibodies against G3P[8] strains.

## Discussion

In this study, we have shown that human G3 rotaviruses became the predominant strains post-Rotarix vaccine introduction in Malawi after replacing G1 and G2 rotaviruses strains. This finding is unique and in contrast with observations seen in most countries, including Australia, Italy, Hungary, Spain, Japan, and Kenya, where the emergence of G3 strains has mostly been due to equine-like G3 strains (Cowley et al. 2016; Dóró et al. 2016; Komoto et al. 2018; Esposito et al. 2019; Luchs et al. 2019; Mwanga et al. 2020). Through whole genome sequencing, we showed that the emergent G3 strains in Malawi were associated with four genotype constellations; G3P[4] and G3P[6] strains with DS-1-like genome segments encoding inner capsid and non-structural proteins, G3P[8] strains with a Wa-like genome segments encoding inner capsid and non-structural proteins, and G3P[4] strains with genome segments encoding DS-1-like inner capsid proteins, DS-1-like non-structural proteins (NSP1, NSP3–NSP5), and a reassortant Wa-like NSP2. Chronologically, these G3 rotaviruses circulated in three phases in Malawi whereby the G3P[4] strains with DS-1-like backbone genes were the first to emerge in November 2017 until August 2018 followed by a sporadic detection of G3P[6] with DS-1-like backbone genes in September 2018, G3P[8] strains with Wa-like backbone genes from December 2018 to August 2019, and, thereafter, reassortant G3P[4] strains with DS-1-like backbone genes and a Wa-like NSP2 (N1) from December 2018 until August 2019. The Wa-like G3P[8] strains predominated in the latter period (2018–9) (Supplementary Table S2). Phylogenetic analysis revealed a wide genetic diversity across all eleven genome segments of the four genotype constellations associated with G3 strains that circulated in Malawi. Our findings also suggest that these G3 strains did not arise from point mutations occurring in the previously circulating strains, but rather through genomic reassortment and importation of strains from other countries. Altogether, these findings highlight the role of genome reassortment in driving rotavirus evolution and human mobility in disseminating rotavirus strains internationally.

Like other segmented viruses, such as influenza (Webster et al. 1992), rotaviruses frequently reassort their genomic segments, which increases their genetic diversity (Martínez-Laso et al. 2009). Although the majority of the human-associated G3 strains have a P[8] and Wa-like genotype constellation (Matthijnssens and Marc 2012), most of the recently emerged G3 rotaviruses possess equine-like as well as DS-1-like rotavirus genome segments (Arana et al. 2016; Cowley et al. 2016; Dóró et al. 2016; Komoto et al. 2018; Esposito et al. 2019; Luchs et al. 2019). Whole genome sequencing of G3 rotavirus strains that were detected from other countries has shown that intergenogroup reassortment events between human and equine rotaviruses drove their emergence (Malasao et al. 2015). In contrast, the emergent G3 strains had typical human Wa-like and DS-1-like genetic constellations. These findings were similar to previous analysis of rotavirus genotypes that circulated in Malawi for over two decades from 1997 that identified a diverse population of rotaviruses (at least 24 G and P genotype combinations) of which WGS revealed that the majority had either a Wa-like or a DS-1-like genetic backbone (Jere et al. 2018; Mhango et al. 2020). The emergent G3 strains in Malawi also had either a Wa-like or a DS-1-like genetic backbone, but phylogenetic analysis revealed up to four lineages in each genome segment (one in all Wa-like and up to three in DS-1-like). These data demonstrate the wide diversity of G3 strains that co-circulated in Malawi within the 2-year study period.

Further phylogenetic analyses of the emergent G3P[4] strains revealed that two populations circulated chronologically in Malawi. The DS-1-like G3P[4] strains that emerged first were genetically closest to sequenced G3 strains from Asia (Pakistan), whereas the reassortant DS-1-like G3P[4] strains that emerged afterwards were genetically distantly related to the former DS-1-like G3P[4] strains in seven genome segments. Our phylogenetic analysis suggested that the reassortant strains did not emerge from the DS-1-like G3P[4] that were first detected in Malawi between 2017 and 2018. In addition, the two G3P[4] populations did not share a recent common ancestor in NSP3, VP1, VP2, and VP4; thus, the reassortant DS-1-like strains were most likely not progenies of the DS-1-like G3P[4] strains that emerged first in Malawi and may have acquired these genome segments from elsewhere or could have been circulating independently at very low frequencies in such a way that could not be picked by our surveillance system. Indeed, three genome segments (NSP5, VP1, and VP4) of the reassortant DS-1-like G3P[4] strains clustered closely with strains from Europe (Czech Republic) instead of other contemporary DS-1-like G3 strains detected in Asia (Pakistan) or DS-1-like G3P[4] and non-G3 strains that circulated previously in Malawi. These findings suggested that these reassortant DS-1-like G3P[4] strains were potentially imported from other countries, although we cannot discount that these strains circulated previously in Malawi as we did not have sufficient sequenced G3 strains before their disappearance in the 1990s. Similarly, phylogenetic analysis of our reassortant DS-1-like G3P[4] showed that their Wa-like NSP2 (N1) genome segments were genetically similar to those from Czech Republic than those from Malawi, while the VP3 genome segments showed a high nucleotide sequence similarity and clustered closely to artiodactyl rotavirus strains characterised elsewhere. Considering our genomic surveillance could not pick up any of these animal-like genome segments, our findings suggested that the VP3 genome segments were not acquired in Malawi; rather, the reassortant strains were potentially seeded into Malawi already having this segment. Therefore, as none of the genome segments of the reassortant DS-1-like G3P[4] resemble strains sequenced from Malawi at any time, it is likely that the reassortant strains containing a Wa-like NSP2 gene acquired their VP7, VP6, NSP1, and NSP4 genome segments from G3P[4] with DS-1-like backbone genes through intragenogroup reassortment elsewhere prior to their introduction into Malawi in 2018. Together, these findings suggest that the typical and reassortant G3 strains that emerged in Malawi were potentially imported, suggesting that importation of rotavirus strains may be an overlooked, but key driver for reseeding genotypes in different countries (Jere et al. 2018; Mhango et al. 2020).

Emerging virus strains have been associated with mutations that render vaccines and other therapies, such as monoclonal antibodies, less effective as seen with the severe acute respiratory syndrome coronavirus 2 (Starr et al. 2021). Previous studies have shown that the VP5* and VP8* outer capsid proteins play a significant role in inducing neutralising antibodies against rotaviruses (Ruggeri and Greenberg 1991; Ludert et al. 2002; Zhao et al. 2015; Park et al. 2021). The VP5* protein of the Malawian G3P[8] strains were 100 per cent conserved when compared to that of Rotarix vaccine, consistent with previous studies that have reported that the VP5* is a highly conserved protein (Mwangi et al. 2020; Rasebotsa et al. 2020). However, we identified E150D non-synonymous substitutions within the VP8*-1 antigenic region and S125N, S131R, and N135D substitutions within VP8*-3 antigenic region when we compared the VP4 (VP8*sub-unit) segments of our G3P[8] strains to that of the strain used in the Rotarix rotavirus vaccine. These substitutions have been associated with Lineage III strains that are currently predominant in eastern and southern African countries, including Malawi, where the Rotarix vaccine is used (Mwangi et al. 2020; Rasebotsa et al. 2020; Maringa et al. 2021). We speculate that the structural changes we observed in the VP8*-1 and VP8*-3 antigenic regions could reduce the binding of the vaccine-induced antibodies thereby reducing the vaccine effectiveness. Further studies are required to investigate the impact of these non-synonymous amino acid changes to generate a complete map of vaccine escape mutants and how they affect antibody neutralisation.

Our study has some limitations. Due to the destruction of the historical stool sample collected through our rotavirus surveillance system as part of the polio containment campaign in Malawi, we included only a few G3P[8] strains from the pre-rotavirus vaccine period sequenced earlier as we were unable to sequence additional pre-vaccine strains. Therefore, although we have shown that the emergent G3P[8] strains in Malawi are unlikely to have emerged from those circulating in Malawi in the pre-vaccination era, we cannot completely rule out that these strains did not emerge from other unsampled strains circulating during this period. We also understand that the contextual rotavirus sequences obtained from GenBank are sparse as sequencing of rotavirus strains is not routinely performed in many countries, which leads to massive surveillance gaps globally. Because of this, we could only infer that the emergent G3 strains in Malawi were imported, but we cannot say with absolute certainty the country of origin for these strains. In addition, we halted our rotavirus surveillance from 2020 to 2021 due to the coronavirus disease 2019 (COVID-19) pandemic, which prevented us from investigating G3 rotaviruses over a longer period after their re-emergence. Regardless, we were able to generate representative G3 strains across the 2 years of rotavirus surveillance that covered the period when G3 genotypes were detected at high frequency.

To our knowledge, this study provides the first comprehensive and systematic genomic characterisation of the re-emerging G3 rotavirus from Africa. Our findings demonstrate that four variants of G3 rotaviruses resembling typical human rotaviruses re-emerged after Rotarix rotavirus vaccine introduction 20 years after disappearing for almost 20 years in Malawi, and their emergence appears to be likely driven by importation from other countries. Our findings highlight the role of human mobility in driving the dissemination and temporal dynamics of circulating rotaviruses internationally and demonstrate the importance of robust rotavirus surveillance and whole genome sequencing to monitor strain dynamics to inform infection prevention and control strategies in high disease–burden settings.

## Methods

### Ethical approval

Informed consent was obtained from all mothers or legal guardians for the children who were involved in this study. This study was conducted according to the guidelines of the Declaration of Helsinki and approved by the Research Ethics Committee of the University of Liverpool, Liverpool, UK (000490), and the National Health Sciences Research Committee, Lilongwe, Malawi (#867).

### Sample collection, rotavirus genotyping, and selection of rotavirus strains

Stool samples were collected from children <5 years old who presented with acute gastroenteritis to the QECH in Blantyre, Malawi, through a rotavirus surveillance platform which has been on-going since 1997 (Mhango et al. 2020). Acute gastroenteritis was defined as the passage of at least three loose or looser-than-normal stools every 24 h for <1 week. The presence of rotaviruses in stool samples was confirmed using Rotaclone^®^ Enzyme Immunosorbent Assay (Rotaclone^®^, Meridian Bioscience, Cincinnati, OH, USA). The VP7 and VP4 genotypes for rotavirus-positive samples were assigned using a multiplex heminested reverse transcriptase–polymerase chain reaction (PCR) as described earlier (Iturriza-Gomara et al. 1999; Mhango et al. 2020). At least one stool specimen containing rotavirus of G3 genotype was selected each calendar month from November 2017 to August 2019 for sequencing (*n* = 27) (Supplementary Fig. 1). Whole genomes for three G3 strains that circulated between 1997 and 1999 collected and sequenced from our previous studies were also analysed (Cunliffe et al. 2010; Jere et al. 2018).

### Extraction of rotavirus dsRNA and synthesis of complementary DNA

Rotavirus dsRNA was extracted and purified as previously described (Jere et al. 2011, 2018). To remove contaminating DNA, the extracted dsRNA was precipitated with lithium chloride (Sigma-Aldrich, Dorset, UK) for 16 h at 4°C and treated with DNase I (Sigma-Aldrich, Dorset, UK) as previously described (Jere et al. 2018). Purified dsRNA was quantified on Qubit 3.0 fluorometer (Life Technologies, CA, USA). A 1 per cent 0.5X Tris borate ethylenediaminetetraacetic acid agarose gel (Sigma-Aldrich, Dorset, UK) stained with SYBR green (Sigma-Aldrich, Dorset, UK) electrophoresis was used to check the integrity of the extracted dsRNA and was visualised on a BioDoc transilluminator. Complementary DNA (cDNA) was synthesised using the Maxima H Minus Double-Stranded cDNA Synthesis Kit (Thermo Fisher Scientific, Waltham, MA, USA), and purification was done using the MSB^®^ Spin PCRapace (Stratec) Purification Kit as previously described (Mwangi et al. 2020; Rasebotsa et al. 2020).

### DNA library preparation and whole genome sequencing

The Nextera XT DNA Library Preparation Kit (Illumina, San Diego, CA, USA) was used to prepare DNA libraries following the manufacturer’s instructions. Briefly, the Nextera^®^ transposome enzyme was used to target genomic DNA which was amplified using a limited cycle PCR. AMPure XP magnetic beads (Beckman Coulter, Pasadena, CA, USA) and 80 per cent alcohol were used to clean up the DNA libraries. Qubit 3.0 fluorometer (Invitrogen, Carlsbad, CA, USA) was used to quantify the cleaned-up DNA libraries. The fragment size and the quality of libraries were assessed using Agilent 2100 BioAnalyzer^®^ (Agilent Technologies, Waldbronn, Germany). Paired end nucleotide sequences were then generated on a MiSeq^®^ sequencer (Illumina, San Diego, CA, USA) at the University of the Free State-Next Generation Sequencing (UFS-NGS) Unit, Bloemfontein, South Africa, as previously described (Mwangi et al. 2020; Rasebotsa et al. 2020).

### Sequence assembly and whole genome genotype determination

We checked the quality of the whole genome sequencing data using FastQC (de Sena Brandine and Smith 2019) and selected samples with quality score >30 for subsequent analysis. Illumina adapter sequences were trimmed from the raw FASTQ sequence data using BBDuk trimmer (version 2) (https://sourceforge.net/projects/bbmap/) embedded in Geneious Prime software (version 2020.1.1) (Kearse et al. 2012). Consensus sequences were generated through mapping of trimmed Illumina reads to prototype rotavirus Wa-like (accession numbers JX406747.1–JX406757.1) and DS-1-like (accession numbers HQ650116.1–HQ650126.1) genogroup reference strains by Geneious Read Mapper (version 6.0.3) with the medium sensitivity and iteratively fine-tuning parameters five times in Geneious Prime software (Kearse et al. 2012). The Geneious consensus tool was used to call the total quality consensus by selecting a 60 per cent highest quality threshold. The gene annotation and prediction tool in Geneious Prime was used to annotate regions of low coverage (<200). To validate the consensus sequences generated by mapping reads to reference sequences, we generated *de novo* assemblies using Iterative Virus Assembler (version 1.0.3) pipeline (Hunt et al. 2015) for comparison. We assigned the genotypes of each assembled genome segment using the Virus Resource Pathogen online server for viral genotyping (Pickett et al. 2012).

### Phylogenetic analysis

To compare our study strains to G3 rotaviruses characterised globally, we obtained the VP7 genome segment of G3 strains from the Virus Variation Resource in the GenBank (Hatcher et al. 2017). We selected genomic segments with a complete ORF and aligned them using MUSCLE (version 3.8.1551) (Edgar 2004). ML phylogenetic trees were then generated in MEGAX (version 10.1.8) with generalised time reversible (GTR) and gamma heterogeneity DNA models. We performed 1,000 bootstraps to assess the reliability of the branching order and partitions in the phylogeny. Annotation of the phylogenetic trees was done using Microreact online server (Argimón et al. 2016).

To explore the genomic diversity of our G3 strains, we used well-known lineage definition frameworks based on the work by Sadiq et al. (2019), Rasebotsa et al. (2020), and Agbemabiese et al. (2019) to assign lineages to the outer capsid protein genes (VP7 and VP4) and genotype 2 genome segments, respectively. Representative reference nucleotide sequences for DS-1-like genotypes for each genome segment were obtained from the Virus Variation Resource in the GenBank database (Hatcher et al. 2017). As there is no well-known lineage definition framework for Wa-like genome segments (genotype 1), global as well as local genotype 1 sequences sampled across the pre-vaccine and post-vaccine periods in Malawi were used as references. The nucleotide sequences for the ORFs of our study strains and reference strains were multiple aligned using MUSCLE (version 3.8.1551) (Edgar 2004). Once aligned, the DNA test models in MEGAX (version 10.1.8) (Tamura, Stecher, and Kumar 2021) were used to identify the optimal evolutionary model that best fit the data for each segment. According to the corrected Akaike Information Criterion as previously described (Kumar et al. 2018), the GTR model with gamma heterogeneity across nucleotide sites was selected and 1,000 bootstraps were used to assess the reliability of the branching order and partitioning during the construction of ML trees (Felsenstein 1988).

### Inference of the time to the most recent common ancestor

To estimate the most recent common ancestor (tMRCA) for each genome segment, we utilised nucleotide sequences of all Wa-like and DS-1-like strains that circulated between 1997 and 2019 in Malawi. We did not do genome-specific analysis for the G3 and P[6] genotypes of the VP7 and VP4, respectively, because we did not have sufficient sequences to conduct the analysis. The reassortant Wa-like NSP2 (N1 genotype) genome segments for the double reassortant G3P[4] study strains were analysed together with other Wa-like NSP2 genome segments. In brief, we aligned genomic segments of previously circulating Wa-like and DS-1-like strains with the Wa-like and DS-1-like G3 segments, respectively, using MAFFT (version 7.487) (Katoh and Standley 2013). The alignments were trimmed at the 3’ and 5’ prime ends to generate sequences of equal lengths while preserving the integrity of the ORF as a pre-analytical process. Trimmed alignments were then used to generate time-resolved trees using tree time (version 0.8.0) (Sagulenko, Puller, and Neher 2018), and the trees were subsequently visualised and annotated using Auspice (version 2.23.0) (Hadfield et al. 2018). We exported the time-resolved trees from Auspice and visualised them using R (version 4.0.3). We used ladderised using the ‘ladderize’ function in ape (version 5.6.2) (Revell 2012) and rooted the tree based on an out-group sequence using the ‘root’ function implemented in phytools (version 0.7.70) (Revell 2012). We estimated the phylogenetic root-to-tip distance based on the sum of branch lengths (transformed to represent time in days) using ‘distRoot’ function in adephylo (version 1.1.11) (Jombart, Balloux, and Dray 2010) and visualised the tree annotated with the genotypes for each strain using the ‘plot.phylo’ function in the ape (version 5.6.2) package (Paradis, Schliep, and Schwartz 2019).

### Structure comparison between the outer capsid VP4 proteins of the G3P[8] and Rotarix vaccine G1P[8] strains

To compare the antigenic sites of the VP4 of G3P[8] and that of Rotarix G1P[8] strains, we aligned their VP4 amino acid sequences using MAFFT (version 7.487) (Katoh and Standley 2013). We targeted the VP5* and VP8* antigenic regions and extracted antigenic sites from the alignments in MEGAX (version 10.1.8) (Tamura, Stecher, and Kumar 2021). To investigate the impact of amino acid substitutions within the antigenic sites on the structural conformation of the neutralising epitopes within the VP4 protein, we selected a representative VP4 amino acid sequences for G3P[8] strains and conducted protein modelling using Modeller (version 9.25) (Eswar et al. 2006). We selected three model structures with the highest Z-dope score and conducted a structural assessment using SWISS-MODEL server. The protein structures were visualised and annotated using PyMOL (version 2.4.1) (DeLano 2002).

## Supplementary Material

vead030_Supp

## Data Availability

The clinical data presented in this study are available on request from the corresponding author. The data are not publicly available due to ethical restrictions. The whole genome sequencing data generated for genome segments utilised in this project were submitted to the National Center for Biotechnology Information (NCBI) database under accession numbers ON791851-792171.
