# Supplementary material for: Comparative whole genome analysis reveals re-emergence of human Wa-like and DS-1-like G3 rotaviruses after Rotarix vaccine introduction in Malawi
Source: Virus Evol. 2023 May 22;9(1):vead030. doi: 10.1093/ve/vead030 (PMC10256189; doi:10.1093/ve/vead030)
Supplement: vead030_Supp [file vead030_supp.zip › suppl_data/Supplementary.pdf]

**Supplementary Table S1. Whole genome Nucleotide and amino acid sequence length for Malawian G3 rotavirus strains.**

| Strain name | Sequence-type | VP7 | VP4 | VP6 | VP1 | VP2 | VP3 | NSP1 | NSP2 | NSP3 | NSP4 | NSP5/6 |
|---|---|---|---|---|---|---|---|---|---|---|---|---|
| **RVA/Human-wt/MWI/BTY22H/2017/G3P[4]** | NT | - | 2359 | 1356 | 3302 | 2684 | 2591 | 1566 | 1059 | 1066 | - | 816 |
| | AA | - | 775 | 397 | 1088 | 879 | 835 | 493 | 317 | 314 | - | 200 |
| **RVA/Human-wt/MWI/BTY22J/2017/G3P[4]** | NT | 1062 | 2359 | 1356 | 3302 | 2684 | 2591 | 1566 | 1059 | 1066 | 751 | 816 |
| | AA | 326 | 775 | 397 | 1088 | 879 | 835 | 493 | 317 | 314 | 184 | 200 |
| **RVA/Human-wt/MWI/BTY232/2017/G3P[4]** | NT | 1062 | 2359 | 1356 | 3302 | 2684 | 2591 | 1566 | 1059 | 1066 | 751 | 816 |
| | AA | 326 | 775 | 397 | 1088 | 879 | 835 | 493 | 317 | 314 | 184 | 200 |
| **RVA/Human-wt/MWI/BTY23I/2018/G3P[4]** | NT | 1062 | 2359 | 1356 | 3302 | 2684 | 2591 | 1566 | 1059 | 1066 | 751 | - |
| | AA | 326 | 775 | 397 | 1088 | 879 | 835 | 493 | 317 | 314 | 184 | - |
| **RVA/Human-wt/MWI/BTY240/2018/G3P[4]** | NT | 1062 | 2359 | 1356 | 3302 | 2684 | 2591 | 1566 | 1059 | 1066 | 751 | 816 |
| | AA | 326 | 775 | 397 | 1088 | 879 | 835 | 493 | 317 | 314 | 184 | 200 |
| **RVA/Human-wt/MWI/BTY24R/2018/G3P[4]** | NT | 1062 | 2359 | 1356 | 3302 | 2684 | 2591 | 1566 | 1059 | 1066 | 751 | 816 |
| | AA | 326 | 775 | 397 | 1088 | 879 | 835 | 493 | 317 | 314 | 184 | 200 |
| **RVA/Human-wt/MWI/BTY250/2018/G3P[4]** | NT | 1062 | 2359 | 1356 | 3302 | 2684 | 2591 | 1566 | 1059 | 1066 | 751 | 816 |
| | AA | 326 | 775 | 397 | 1088 | 879 | 835 | 493 | 317 | 314 | 184 | 200 |
| **RVA/Human-wt/MWI/BTY25L/2018/G3P[4]** | NT | 1062 | 2359 | 1356 | 3302 | 2684 | 2591 | 1566 | 1059 | 1066 | 751 | 816 |
| | AA | 326 | 775 | 397 | 1088 | 879 | 835 | 493 | 317 | 314 | 184 | 200 |
| **RVA/Human-wt/MWI/BTY260/2018/G3P[4]** | NT | 1062 | 2359 | 1356 | 3302 | 2684 | 2591 | 1566 | 1059 | 1066 | 751 | 816 |
| | AA | 326 | 775 | 397 | 1088 | 879 | 835 | 493 | 317 | 314 | 184 | 200 |
| **RVA/Human-wt/MWI/BTY26H/2018/G3P[4]** | NT | 1062 | 2359 | 1356 | 3302 | 2684 | 2591 | 1566 | 1059 | 1066 | 751 | 816 |
| | AA | 326 | 775 | 397 | 1088 | 879 | 835 | 493 | 317 | 314 | 184 | 200 |
| **RVA/Human-wt/MWI/BTY27C/2018/G3P[4]** | NT | 1062 | 2359 | 1356 | 3302 | 2684 | 2591 | 1566 | 1059 | 1066 | 751 | 816 |
| | AA | 326 | 775 | 397 | 1088 | 879 | 835 | 493 | 317 | 314 | 184 | 200 |
| **RVA/Human-wt/MWI/BTY27S/2018/G3P[4]** | NT | 1062 | 2359 | 1356 | 3302 | 2684 | 2591 | 1566 | 1059 | 1066 | 751 | 816 |
| | AA | 326 | 775 | 397 | 1088 | 879 | 835 | 493 | 317 | 314 | 184 | 200 |
| **RVA/Human-wt/MWI/BTY296/2018/G3P[4]** | NT | 1062 | 2359 | 1356 | 3302 | 2684 | 2591 | 1566 | 1059 | 1066 | 751 | 816 |
| | AA | 326 | 775 | 397 | 1088 | 879 | 835 | 493 | 317 | 314 | 184 | 200 |
| **RVA/Human-wt/MWI/BTY29D/2018/G3P[4]** | NT | 1062 | 2359 | 1356 | 3302 | 2684 | 2591 | 1566 | 1059 | 1066 | 751 | 816 |
| | AA | 326 | 775 | 397 | 1088 | 879 | 835 | 493 | 317 | 314 | 184 | 200 |
| **RVA/Human-wt/MWI/BTY29E/2018/G3P[4]** | NT | 1062 | 2359 | 1356 | 3302 | 2684 | 2591 | 1566 | 1059 | 1066 | 751 | - |
| | AA | 326 | 775 | 397 | 1088 | 879 | 835 | 493 | 317 | 314 | 184 | - |
| **RVA/Human-wt/MWI/BTY2A2/2018/G3P[4]** | NT | 1062 | - | 1356 | 3302 | 2684 | 2591 | 1566 | 1059 | 1066 | 751 | - |
| | AA | 326 | - | 397 | 1088 | 879 | 835 | 493 | 317 | 314 | 184 | - |
| **RVA/Human-wt/MWI/BTY2BG/2018/G3P[4]** | NT | 1062 | 2359 | 1356 | 3302 | 2684 | 2591 | 1566 | 1059 | 1066 | 751 | 816 |
| | AA | 326 | 775 | 397 | 1088 | 879 | 835 | 493 | 317 | 314 | 184 | 200 |
| **RVA/Human-wt/MWI/BTY2EP/2019/G3P[4]** | NT | 1062 | 2359 | 1356 | 3302 | 2684 | 2591 | 1566 | 1059 | 1066 | 751 | - |
| | AA | 326 | 775 | 397 | 1088 | 879 | 835 | 493 | 317 | 314 | 184 | - |
| **RVA/Human-wt/MWI/CHX11Q/2019/G3P[4]** | NT | 1062 | 2359 | 1356 | 3302 | 2684 | 2591 | 1566 | 1059 | 1066 | 751 | 816 |
| | AA | 326 | 775 | 397 | 1088 | 879 | 835 | 493 | 317 | 314 | 184 | 200 |
| **RVA/Human-wt/MWI/CHX11X/2019/G3P[4]** | NT | 1062 | 2359 | 1356 | 3302 | 2684 | 2591 | 1566 | 1059 | 1066 | 751 | - |
| | AA | 326 | 775 | 397 | 1088 | 879 | 835 | 493 | 317 | 314 | 184 | 200 |
| **RVA/Human-wt/MWI/BTY2CM/2018/G3P[6]** | NT | 1062 | 2359 | 1356 | 3302 | 2684 | 2591 | 1566 | 1059 | 1066 | 751 | 816 |
| | AA | 326 | 775 | 397 | 1088 | 879 | 835 | 493 | 317 | 314 | 184 | 200 |
| **RVA/Human-wt/MWI/CBTY2BD/2018/G3P[8]** | NT | 1062 | 2359 | 1356 | 3302 | 2729 | 2591 | 1567 | 1059 | 1074 | 750 | 664 |
| | AA | 326 | 775 | 397 | 1088 | 894 | 835 | 493 | 317 | 314 | 184 | 197 |
| **RVA/Human-wt/MWI/BTY2EM/2019/G3P[8]** | NT | 1062 | 2359 | 1356 | 3302 | 2729 | 2591 | 1567 | 1059 | 1074 | 750 | 664 |
| | AA | 326 | 775 | 397 | 1088 | 894 | 835 | 493 | 317 | 314 | 184 | 197 |

| | | | | | | | | | | | | |
|---|---|---|---|---|---|---|---|---|---|---|---|---|
| **RVA/Human-wt/MWI/BTY2GA/2019/G3P[8]** | NT | 1062 | 2359 | 1356 | 3302 | 2729 | 2591 | 1567 | 1059 | 1074 | 750 | 664 |
| | AA | 326 | 775 | 397 | 1088 | 894 | 835 | 493 | 317 | 314 | 184 | 197 |
| **RVA/Human-wt/MWI/BTY2GC/2019/G3P[8]** | NT | 1062 | 2359 | 1356 | 3302 | 2729 | 2591 | 1567 | 1059 | 1074 | 750 | 664 |
| | AA | 326 | 775 | 397 | 1088 | 894 | 835 | 493 | 317 | 314 | 184 | 197 |
| **RVA/Human-wt/MWI/CHX11U/2019/G3P[8]** | NT | 1062 | 2359 | 1356 | 3302 | 2729 | 2591 | 1567 | 1059 | 1074 | 750 | 664 |
| | AA | 326 | 775 | 397 | 1088 | 894 | 835 | 493 | 317 | 314 | 184 | 197 |
| **RVA/Human-wt/MWI/CHX11S/2019/G3P[8]** | NT | 1062 | 2359 | 1356 | 3302 | 2729 | 2591 | 1567 | 1059 | 1074 | 750 | 664 |
| | AA | 326 | 775 | 397 | 1088 | 894 | 835 | 493 | 317 | 314 | 184 | 197 |

**Supplementary Table S2**. **Chronological presentation of G3 strains utilized in the study.** The nomenclature of all the rotavirus strains indicates the rotavirus group, species where the strain was isolated, name of the country where the strain was originally isolated, the common name, year of isolation and the genotypes for genome segment 4 and 9 as proposed by the *Rotavirus Classification Working Group* (RCWG) (Matthijnssens et al. 2008).

| G3P[4] STRAINS | Month | Year | VP7 | VP4 | VP6 | VP1 | VP2 | VP3 | NSP1 | NSP2 | NSP3 | NSP4 | NSP5 | |
|---|---|---|---|---|---|---|---|---|---|---|---|---|---|---|
| RVA/Human-wt/MWI/BTY22H/2017/G3P[4] | November | 2017 | - | P[4] | I2 | R2 | C2 | M2 | A2 | N2 | T2 | - | H2 | G3P[4] DS-1 like backbone genes |
| RVA/Human-wt/MWI/BTY22J/2017/G3P[4] | November | 2017 | G3 | P[4] | I2 | R2 | C2 | M2 | A2 | N2 | T2 | E2 | H2 | G3P[4] DS-1 like backbone genes |
| RVA/Human-wt/MWI/BTY232/2017/G3P[4] | December | 2017 | G3 | P[4] | I2 | R2 | C2 | M2 | A2 | N2 | T2 | E2 | H2 | G3P[4] DS-1 like backbone genes |
| RVA/Human-wt/MWI/BTY23I/2018/G3P[4] | January | 2018 | G3 | P[4] | I2 | R2 | C2 | M2 | A2 | N2 | T2 | E2 | - | G3P[4] DS-1 like backbone genes |
| RVA/Human-wt/MWI/BTY240/2018/G3P[4] | January | 2018 | G3 | P[4] | I2 | R2 | C2 | M2 | A2 | N2 | T2 | E2 | H2 | G3P[4] DS-1 like backbone genes |
| RVA/Human-wt/MWI/BTY24R/2018/G3P[4] | February | 2018 | G3 | P[4] | I2 | R2 | C2 | M2 | A2 | N2 | T2 | E2 | H2 | G3P[4] DS-1 like backbone genes |
| RVA/Human-wt/MWI/BTY25L/2018/G3P[4] | March | 2018 | G3 | P[4] | I2 | R2 | C2 | M2 | A2 | N2 | T2 | E2 | H2 | G3P[4] DS-1 like backbone genes |
| RVA/Human-wt/MWI/BTY25Q/2018/G3P[4] | March | 2018 | G3 | P[4] | I2 | R2 | C2 | M2 | A2 | N2 | T2 | E2 | H2 | G3P[4] DS-1 like backbone genes |
| RVA/Human-wt/MWI/BTY26H/2018/G3P[4] | April | 2018 | G3 | P[4] | I2 | R2 | C2 | M2 | A2 | N2 | T2 | E2 | H2 | G3P[4] DS-1 like backbone genes |
| RVA/Human-wt/MWI/BTY26Q/2018/G3P[4] | April | 2018 | G3 | P[4] | I2 | R2 | C2 | M2 | A2 | N2 | T2 | E2 | H2 | G3P[4] DS-1 like backbone genes |
| RVA/Human-wt/MWI/BTY27C/2018/G3P[4] | May | 2018 | G3 | P[4] | I2 | R2 | C2 | M2 | A2 | N2 | T2 | E2 | H2 | G3P[4] DS-1 like backbone genes |
| RVA/Human-wt/MWI/BTY27S/2018/G3P[4] | May | 2018 | G3 | P[4] | I2 | R2 | C2 | M2 | A2 | N2 | T2 | E2 | H2 | G3P[4] DS-1 like backbone genes |
| RVA/Human-wt/MWI/BTY296/2018/G3P[4] | June | 2018 | G3 | P[4] | I2 | R2 | C2 | M2 | A2 | N2 | T2 | E2 | H2 | G3P[4] DS-1 like backbone genes |
| RVA/Human-wt/MWI/BTY29D/2018/G3P[4] | July | 2018 | G3 | P[4] | I2 | R2 | C2 | M2 | A2 | N2 | T2 | E2 | H2 | G3P[4] DS-1 like backbone genes |
| RVA/Human-wt/MWI/BTY29E/2018/G3P[4] | July | 2018 | G3 | P[4] | I2 | R2 | C2 | M2 | A2 | N2 | T2 | E2 | - | G3P[4] DS-1 like backbone genes |
| RVA/Human-wt/MWI/BTY2A2/2018/G3P[4] | August | 2018 | G3 | - | I2 | R2 | C2 | M2 | A2 | N2 | T2 | E2 | - | G3P[4] DS-1 like backbone genes |
| RVA/Human-wt/MWI/BTY2CM/2018/G3P[6] | September | 2018 | G3 | P[6] | I2 | R2 | C2 | M2 | A2 | N2 | T2 | E2 | H2 | G3P[6] DS-1 like backbone genes |
| RVA/Human-wt/MWI/BTY2BD/2018/G3P[8] | December | 2018 | G3 | P[8] | I1 | R1 | C1 | M1 | A1 | N1 | T1 | E1 | H1 | G3P[8] Wa-like backbone genes |
| RVA/Human-wt/MWI/BTY2BG/2018/G3P[4] | December | 2018 | G3 | P[4] | I2 | R2 | C2 | M2 | A2 | N1 | T2 | E2 | H2 | G3P[4] DS-1 like backbone genes and reassortant N1 NSP2 |
| RVA/Human-wt/MWI/BTY2EM/2019/G3P[8] | March | 2019 | G3 | P[8] | I1 | R1 | C1 | M1 | A1 | N1 | T1 | E1 | H1 | G3P[8] Wa-like backbone genes |
| RVA/Human-wt/MWI/BTY2GA/2019/G3P[8] | April | 2019 | G3 | P[8] | I1 | R1 | C1 | M1 | A1 | N1 | T1 | E1 | H1 | G3P[8] Wa-like backbone genes |
| RVA/Human-wt/MWI/BTY2GC/2019/G3P[8] | April | 2019 | G3 | P[8] | I1 | R1 | C1 | M1 | A1 | N1 | T1 | E1 | H1 | G3P[8] Wa-like backbone genes |
| RVA/Human-wt/MWI/BTY2EP/2019/G3P[4] | May | 2019 | G3 | P[4] | I2 | R2 | C2 | M2 | A2 | N1 | T2 | E2 | - | G3P[4] DS-1 like backbone genes and reassortant N1 NSP2 |
| RVA/Human-wt/MWI/CHX11Q/2019/G3P[4] | July | 2019 | G3 | P[4] | I2 | R2 | C2 | M2 | A2 | N1 | T2 | E2 | H2 | G3P[4] DS-1 like backbone genes and reassortant N1 NSP2 |
| RVA/Human-wt/MWI/CHX11S/2019/G3P[8] | August | 2019 | G3 | P[8] | I1 | R1 | C1 | M1 | A1 | N1 | T1 | E1 | H1 | G3P[8] Wa-like backbone genes |
| RVA/Human-wt/MWI/CHX11U/2019/G3P[8] | August | 2019 | G3 | P[8] | I1 | R1 | C1 | M1 | A1 | N1 | T1 | E1 | H1 | G3P[8] Wa-like backbone genes |
| RVA/Human-wt/MWI/CHX11X/2019/G3P[4] | August | 2019 | G3 | P[4] | I2 | R2 | C2 | M2 | A2 | N1 | T2 | E2 | - | G3P[4] DS-1 like backbone genes and reassortant N1 NSP2 |

**Supplementary Table S3**: **Lineages for Wa-like (green) and DS-1-like (red) genome segments associated with re-emergent G3 rotavirus strains in Malawi**. There is no lineage framework available for Wa-like genome segments hence the only available Wa-like reference sequences that were assigned lineages previously were utilised to assign lineages to VP4 and VP7 encoding genome segments of the Malawian G3 strains.

<table>
<tr><th></th><th>VP7</th><th colspan="2">VP4</th><th>VP6</th><th>VP1</th><th>VP2</th><th colspan="2">VP3</th><th>NSP1</th><th>NSP2</th><th>NSP3</th><th>NSP4</th><th>NSP5</th></tr>
<tr><td>G3P[4]</td><td>III</td><td>IVa</td><td>V</td><td>V</td><td>V</td><td>IVa</td><td>VII</td><td>VI</td><td>IVa</td><td>V</td><td>V</td><td>VI</td><td>IVa</td></tr>
<tr><td>G3P[6]</td><td>III</td><td colspan="2">Ia</td><td>V</td><td>V</td><td>IVa</td><td colspan="2">VII</td><td>IVa</td><td>V</td><td>V</td><td>VI</td><td>IVa</td></tr>
<tr><td>G3P[8]</td><td>III</td><td colspan="2">III</td><td>-</td><td>-</td><td>-</td><td colspan="2">-</td><td>-</td><td>-</td><td>-</td><td>-</td><td>-</td></tr>
</table>

**Supplementary Table S4. Alignment of antigenic residues of VP4 protein between the P[8] component of Rotarix and VP4 component of Malawian G3 strains**. Antigenic residues are divided into four antigenic epitopes for VP8* (8-1, 8-2, 8-3 and 8-4) and five antigenic epitopes for VP5* (5-1, 5-2, 5-3, 5-4, and 5-5). Amino acid changes that have been shown to escape neutralisation with monoclonal antibodies are indicated with a black dot

| | | | 8*-1 | | | | | | | | | | | 8*-2 | | 8*-3 | | | | | | | | | 8*-4 | | | 5*-1 | | | | | | | | 5*-2 | 5*-3 | 5*-4 | 5*-5 |
|---|---|---|---|---|---|---|---|---|---|---|---|---|---|---|---|---|---|---|---|---|---|---|---|---|---|---|---|---|---|---|---|---|---|---|---|---|---|---|---|
| | | | ● | ● | ● | ● | ● | ● | | | ● | | | ● | ● | | ● | | ● | | | ● | ● | ● | ● | ● | ● | ● | ● | ● | ● | ● | ● | ● | ● | ● | ● | ● | ● |
| **Strain** | **Genotype** | **Lineage** | **100** | **146** | **148** | **150** | **188** | **190** | **192** | **193** | **194** | **195** | **196** | **180** | **183** | **113** | **114** | **115** | **116** | **125** | **131** | **132** | **133** | **135** | **87** | **88** | **89** | **384** | **386** | **388** | **393** | **394** | **398** | **440** | **441** | **434** | **459** | **429** | **306** |
| **ROTARIX** | P[8] | I | D | S | Q | E | S | T | N | L | N | N | I | T | A | N | P | V | D | S | S | N | D | N | N | T | N | Y | F | I | W | P | G | R | T | P | E | L | L |
| **BTY2GC** | P[8] | III | * | * | * | D | * | * | * | * | * | * | * | * | * | * | * | * | * | N | R | * | * | D | * | * | * | * | * | * | * | * | * | * | * | * | * | * | * |
| **BTY2GA** | P[8] | III | * | * | * | D | * | * | * | * | * | * | * | * | * | * | * | * | * | N | R | * | * | D | * | * | * | * | * | * | * | * | * | * | * | * | * | * | * |
| **BTY2BD** | P[8] | III | * | * | * | D | * | * | * | * | * | * | * | * | * | * | * | * | * | N | R | * | * | D | * | * | * | * | * | * | * | * | * | * | * | * | * | * | * |
| **BTY2EM** | P[8] | III | * | * | * | D | * | * | * | * | * | * | * | * | * | * | * | * | * | N | R | * | * | D | * | * | * | * | * | * | * | * | * | * | * | * | * | * | * |
| **CHX11U** | P[8] | III | * | * | * | D | * | * | * | * | * | * | * | * | * | * | * | * | * | N | R | * | * | D | * | * | * | * | * | * | * | * | * | * | * | * | * | * | * |
| **CHX11S** | P[8] | III | * | * | * | D | * | * | * | * | * | * | * | * | * | * | * | * | * | N | R | * | * | D | * | * | * | * | * | * | * | * | * | * | * | * | * | * | * |
| **BTY2EP** | P[4] | IVa | * | * | * | D | * | * | D | * | * | N | * | * | * | S | * | T | N | N | E | * | S | D | * | * | D | * | * | L | * | * | * | * | * | * | * | * | * |
| **BTY2BG** | P[4] | IVa | * | * | * | D | * | * | D | * | * | N | * | * | * | S | * | T | N | N | E | * | S | D | * | * | D | * | * | L | * | * | * | * | * | * | * | * | * |
| **BTY29D** | P[4] | V | * | * | * | D | * | * | D | * | * | N | * | * | * | S | Q | T | N | N | E | * | S | D | * | A | D | * | * | L | * | * | * | * | * | * | * | * | * |
| **BTY22H** | P[4] | V | * | * | * | D | * | * | D | * | * | N | * | * | * | S | Q | T | N | N | E | * | S | D | * | * | D | * | * | L | * | * | * | * | * | * | * | * | * |
| **BTY2CM** | P[6] | Ia | * | * | S | * | * | * | * | * | S | * | V | * | * | T | N | Q | S | T | E | * | N | S | T | N | Q | * | * | * | * | * | * | * | * | * | * | * | * |

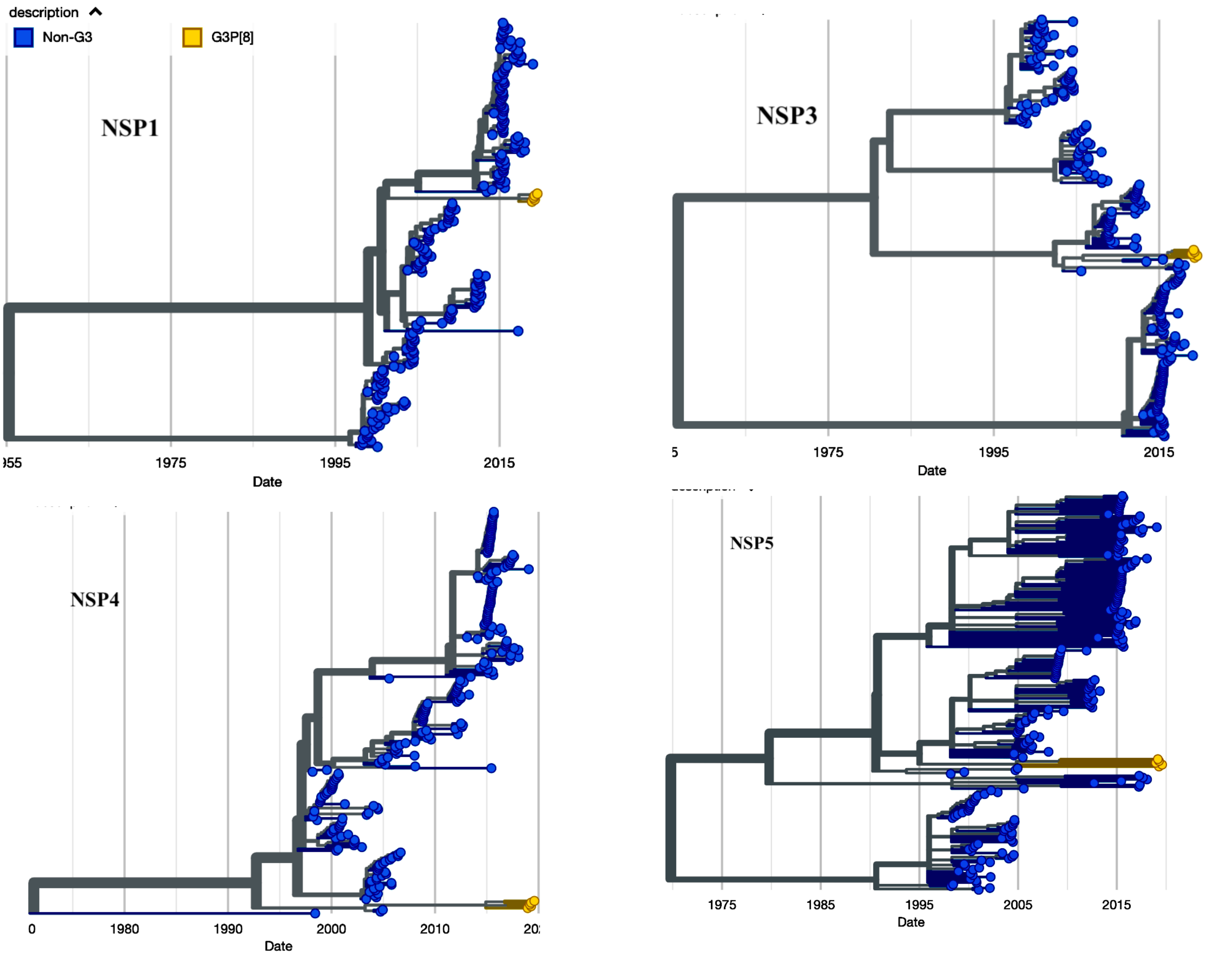

description
Non-G3
G3P[8]
NSP1
955
1975
1995
2015
Date
NSP3
5
1975
1995
2015
Date
NSP4
0
1980
1990
2000
2010
20
Date
NSP5
1975
1985
1995
2005
2015
Date

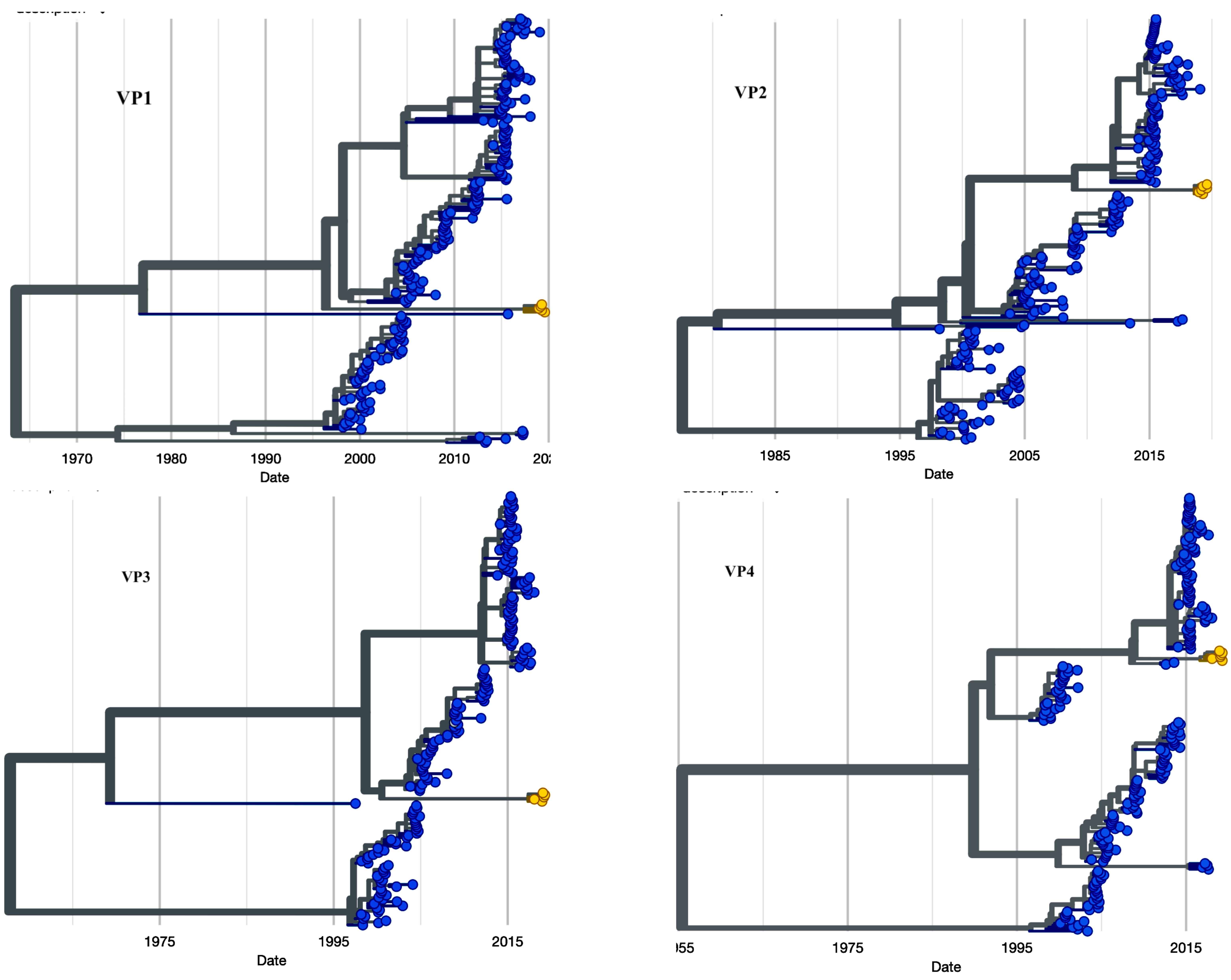
VP1
1970
1980
1990
2000
2010
Date
VP2
1985
1995
2005
2015
Date
VP3
1975
1995
2015
Date
VP4
1975
1995
2015
Date

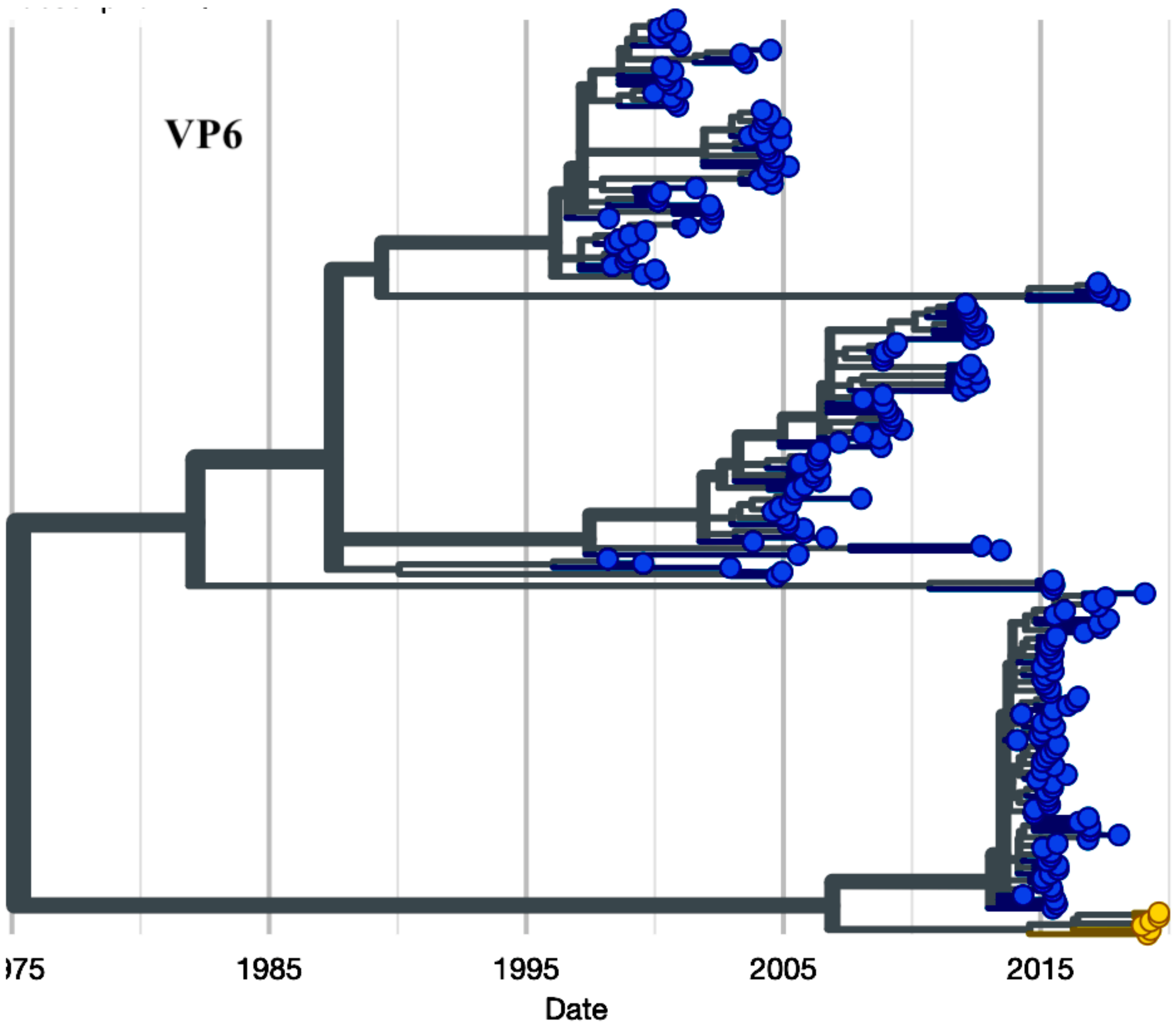


**Supplementary Figure S1. Time resolved phylogenetic trees generated using Nexstrain for all Wa-like genome segments detected in Malawi from 1997 to 2019 and Wa-like genome segments associated with re-emergent G3 strains**. The previously circulating Wa-like segments were used to estimate time to the most recent ancestor for the Wa-like G3 strains in relation to locally circulating Wa-like genome segments in Blantyre, Malawi. Genome segments associated with G3 rotavirus strains are annotated in yellow tips while those associated with non-G3 strains are annotated in blue tips. The VP4 segment only includes the P[8] genotype associated with G3 as well as other non-G3 strains characterized between 1997 and 2019. The rest of the genome segments are genotype 1 (Wa-like) only.

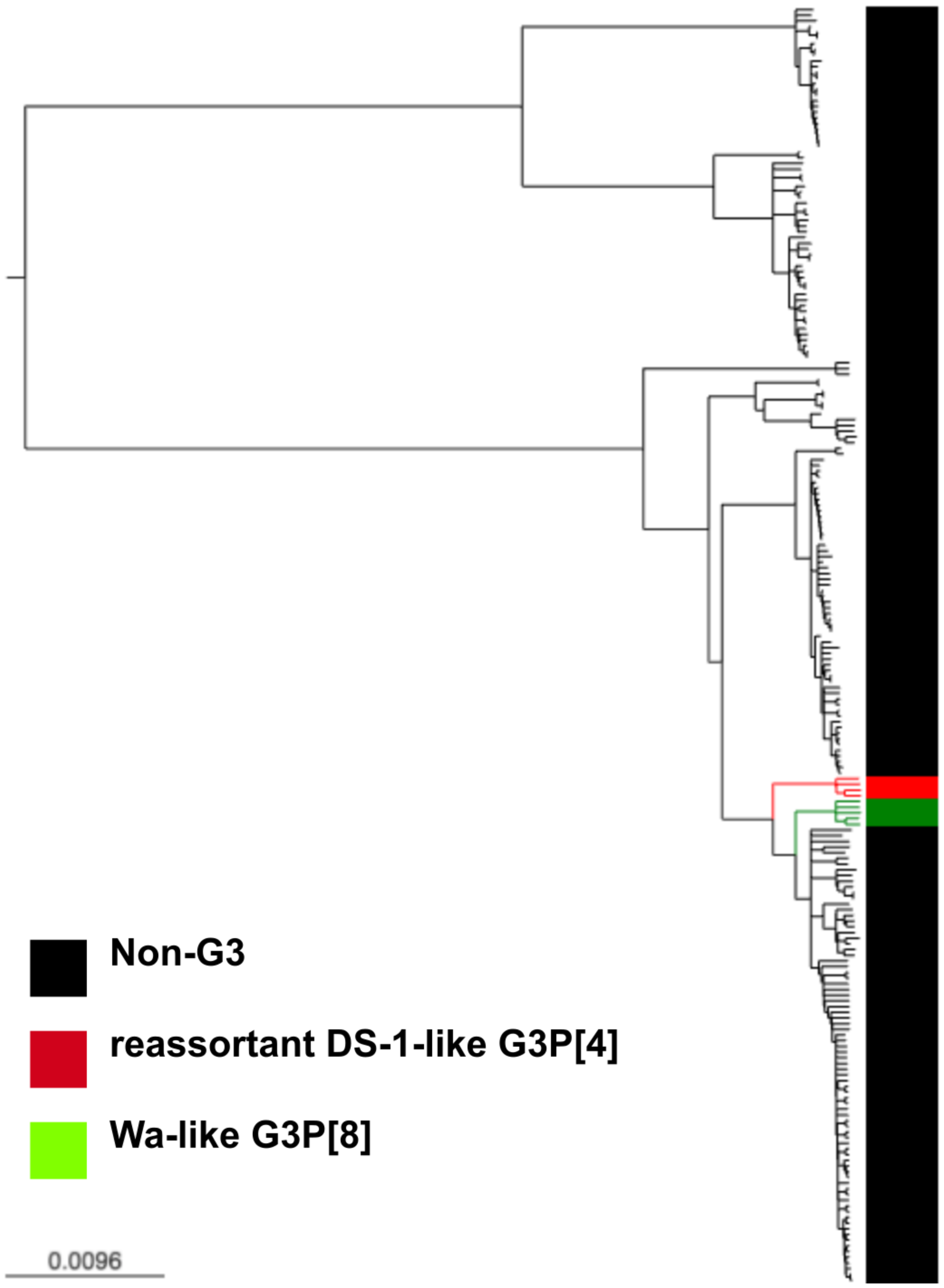


**Supplementary Figure S2**. **Maximum likelihood (ML) phylogenetic tree of all N1 NSP2 genotypes associated with G3 as well as non-G3 strains characterised in Malawi.** Only strains with a complete open reading frame were included in the analysis. The GTR evolutionary model with Gamma heterogeneity across nucleotide sites was used for phylogenetic inference. Bootstrap values ≥70% are shown adjacent to each branch node. The trees were out grouped at RVA/Pigeon-wt/JPN/P0-13/1989/G18P[17] but it was removed in the final tree for better visualization. Malawian Wa-like G3P[8] and reassortant DS-1-like G3P[4] strains are denoted by green and red colours respectively.

a. VP7

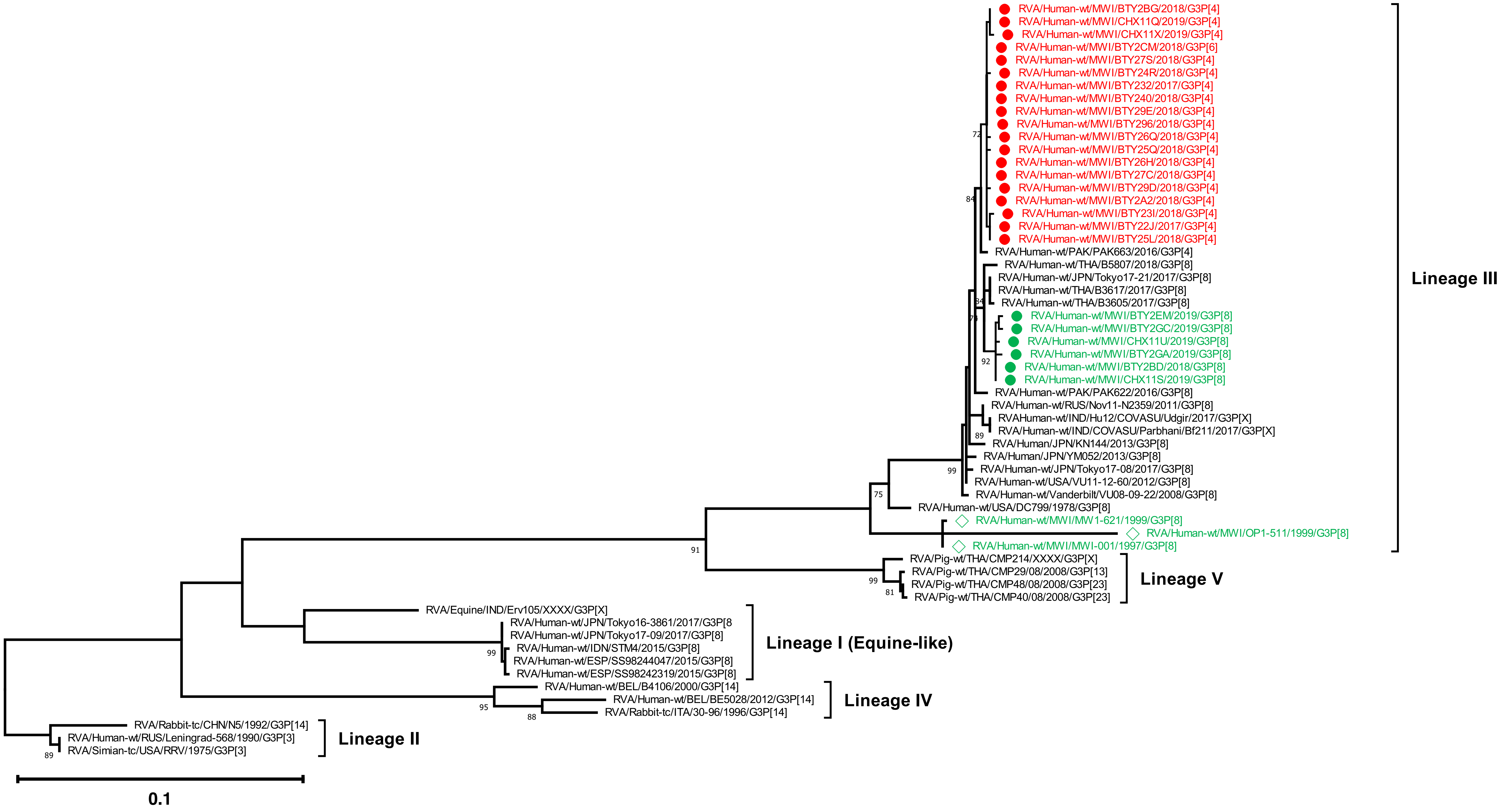
RVA/Human-wt/MWI/BTY2BG/2018/G3P[4]
RVA/Human-wt/MWI/CHX11Q/2019/G3P[4]
RVA/Human-wt/MWI/CHX11X/2019/G3P[4]
RVA/Human-wt/MWI/BTY2CM/2018/G3P[6]
RVA/Human-wt/MWI/BTY27S/2018/G3P[4]
RVA/Human-wt/MWI/BTY24R/2018/G3P[4]
RVA/Human-wt/MWI/BTY232/2017/G3P[4]
RVA/Human-wt/MWI/BTY240/2018/G3P[4]
RVA/Human-wt/MWI/BTY29E/2018/G3P[4]
RVA/Human-wt/MWI/BTY296/2018/G3P[4]
RVA/Human-wt/MWI/BTY26Q/2018/G3P[4]
RVA/Human-wt/MWI/BTY25Q/2018/G3P[4]
RVA/Human-wt/MWI/BTY26H/2018/G3P[4]
RVA/Human-wt/MWI/BTY27C/2018/G3P[4]
RVA/Human-wt/MWI/BTY29D/2018/G3P[4]
RVA/Human-wt/MWI/BTY2A2/2018/G3P[4]
RVA/Human-wt/MWI/BTY23I/2018/G3P[4]
RVA/Human-wt/MWI/BTY22J/2017/G3P[4]
RVA/Human-wt/MWI/BTY25L/2018/G3P[4]
RVA/Human-wt/PAK/PAK663/2016/G3P[4]
RVA/Human-wt/THA/B5807/2018/G3P[8]
RVA/Human-wt/JPN/Tokyo17-21/2017/G3P[8]
RVA/Human-wt/THA/B3617/2017/G3P[8]
RVA/Human-wt/THA/B3605/2017/G3P[8]
RVA/Human-wt/MWI/BTY2EM/2019/G3P[8]
RVA/Human-wt/MWI/BTY2GC/2019/G3P[8]
RVA/Human-wt/MWI/CHX11U/2019/G3P[8]
RVA/Human-wt/MWI/BTY2GA/2019/G3P[8]
RVA/Human-wt/MWI/BTY2BD/2018/G3P[8]
RVA/Human-wt/MWI/CHX11S/2019/G3P[8]
RVA/Human-wt/PAK/PAK622/2016/G3P[8]
RVA/Human-wt/RUS/Nov11-N2359/2011/G3P[8]
RVAHuman-wt/IND/Hu12/COVASU/Udgir/2017/G3P[X]
RVA/Human-wt/IND/COVASU/Parbhani/Bf211/2017/G3P[X]
RVA/Human/JPN/KN144/2013/G3P[8]
RVA/Human/JPN/YM052/2013/G3P[8]
RVA/Human-wt/JPN/Tokyo17-08/2017/G3P[8]
RVA/Human-wt/USA/VU11-12-60/2012/G3P[8]
RVA/Human-wt/Vanderbilt/VU08-09-22/2008/G3P[8]
RVA/Human-wt/USA/DC799/1978/G3P[8]
RVA/Human-wt/MWI/MW1-621/1999/G3P[8]
RVA/Human-wt/MWI/OP1-511/1999/G3P[8]
RVA/Human-wt/MWI/MWI-001/1997/G3P[8]
Lineage III
RVA/Pig-wt/THA/CMP214/XXXX/G3P[X]
RVA/Pig-wt/THA/CMP29/08/2008/G3P[13]
RVA/Pig-wt/THA/CMP48/08/2008/G3P[23]
RVA/Pig-wt/THA/CMP40/08/2008/G3P[23]
Lineage V
RVA/Equine/IND/Erv105/XXXX/G3P[X]
RVA/Human-wt/JPN/Tokyo16-3861/2017/G3P[8
RVA/Human-wt/JPN/Tokyo17-09/2017/G3P[8]
RVA/Human-wt/IDN/STM4/2015/G3P[8]
RVA/Human-wt/ESP/SS98244047/2015/G3P[8]
RVA/Human-wt/ESP/SS98242319/2015/G3P[8]
Lineage I (Equine-like)
RVA/Human-wt/BEL/B4106/2000/G3P[14]
RVA/Human-wt/BEL/BE5028/2012/G3P[14]
RVA/Rabbit-tc/ITA/30-96/1996/G3P[14]
Lineage IV
RVA/Rabbit-tc/CHN/N5/1992/G3P[14]
RVA/Human-wt/RUS/Leningrad-568/1990/G3P[3]
RVA/Simian-tc/USA/RRV/1975/G3P[3]
Lineage II
72
84
84
73
92
89
99
75
91
99
81
99
95
88
89
0.1

b. VP6

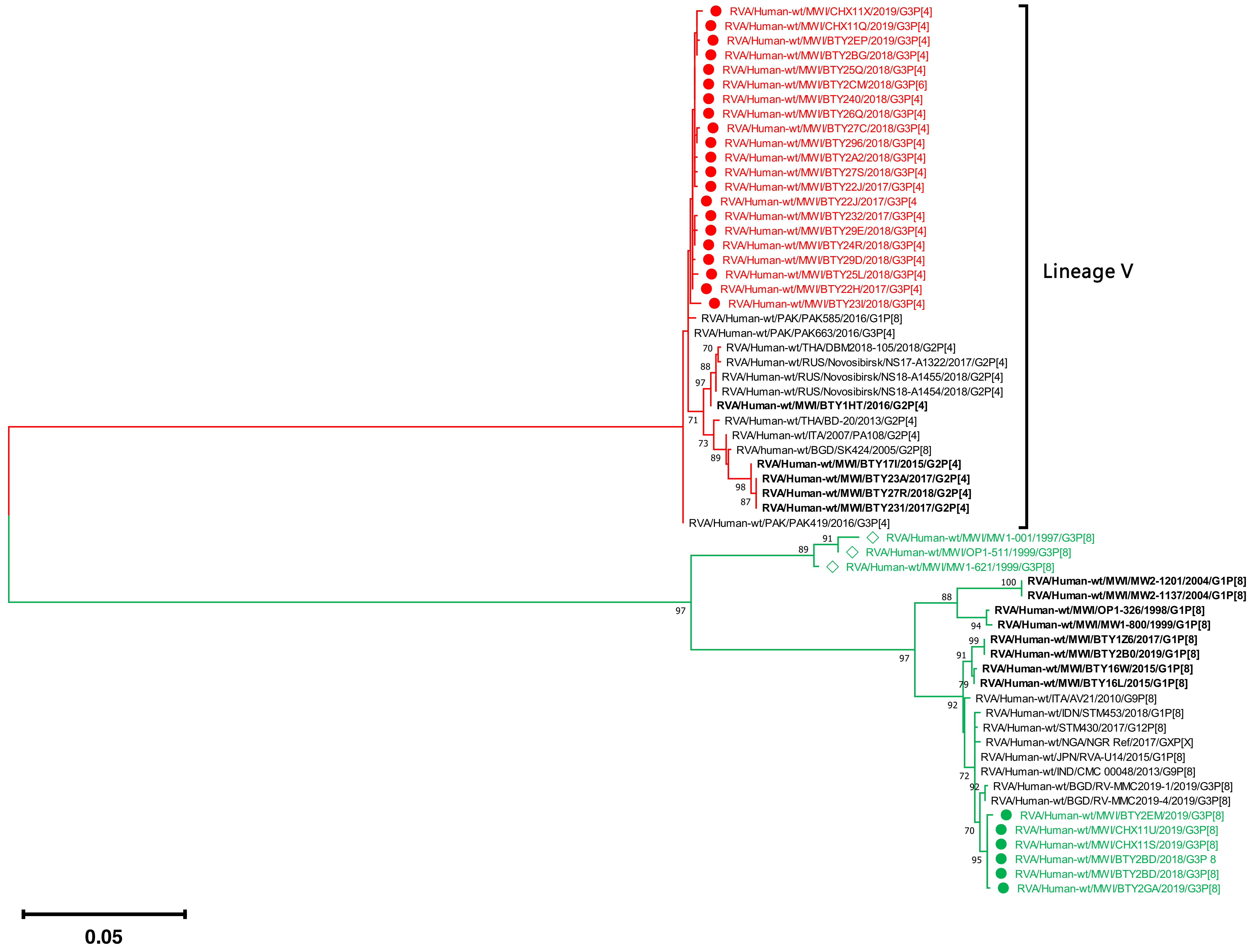
RVA/Human-wt/MWI/CHX11X/2019/G3P[4]
RVA/Human-wt/MWI/CHX11Q/2019/G3P[4]
RVA/Human-wt/MWI/BTY2EP/2019/G3P[4]
RVA/Human-wt/MWI/BTY2BG/2018/G3P[4]
RVA/Human-wt/MWI/BTY25Q/2018/G3P[4]
RVA/Human-wt/MWI/BTY2CM/2018/G3P[6]
RVA/Human-wt/MWI/BTY240/2018/G3P[4]
RVA/Human-wt/MWI/BTY26Q/2018/G3P[4]
RVA/Human-wt/MWI/BTY27C/2018/G3P[4]
RVA/Human-wt/MWI/BTY296/2018/G3P[4]
RVA/Human-wt/MWI/BTY2A2/2018/G3P[4]
RVA/Human-wt/MWI/BTY27S/2018/G3P[4]
RVA/Human-wt/MWI/BTY22J/2017/G3P[4]
RVA/Human-wt/MWI/BTY22J/2017/G3P[4
RVA/Human-wt/MWI/BTY232/2017/G3P[4]
RVA/Human-wt/MWI/BTY29E/2018/G3P[4]
RVA/Human-wt/MWI/BTY24R/2018/G3P[4]
RVA/Human-wt/MWI/BTY29D/2018/G3P[4]
RVA/Human-wt/MWI/BTY25L/2018/G3P[4]
RVA/Human-wt/MWI/BTY22H/2017/G3P[4]
RVA/Human-wt/MWI/BTY23I/2018/G3P[4]
RVA/Human-wt/PAK/PAK585/2016/G1P[8]
RVA/Human-wt/PAK/PAK663/2016/G3P[4]
RVA/Human-wt/THA/DBM2018-105/2018/G2P[4]
RVA/Human-wt/RUS/Novosibirsk/NS17-A1322/2017/G2P[4]
RVA/Human-wt/RUS/Novosibirsk/NS18-A1455/2018/G2P[4]
RVA/Human-wt/RUS/Novosibirsk/NS18-A1454/2018/G2P[4]
RVA/Human-wt/MWI/BTY1HT/2016/G2P[4]
RVA/Human-wt/THA/BD-20/2013/G2P[4]
RVA/Human-wt/ITA/2007/PA108/G2P[4]
RVA/human-wt/BGD/SK424/2005/G2P[8]
RVA/Human-wt/MWI/BTY17I/2015/G2P[4]
RVA/Human-wt/MWI/BTY23A/2017/G2P[4]
RVA/Human-wt/MWI/BTY27R/2018/G2P[4]
RVA/Human-wt/MWI/BTY231/2017/G2P[4]
RVA/Human-wt/PAK/PAK419/2016/G3P[4]
Lineage V
RVA/Human-wt/MWI/MW1-001/1997/G3P[8]
RVA/Human-wt/MWI/OP1-511/1999/G3P[8]
RVA/Human-wt/MWI/MW1-621/1999/G3P[8]
RVA/Human-wt/MWI/MW2-1201/2004/G1P[8]
RVA/Human-wt/MWI/MW2-1137/2004/G1P[8]
RVA/Human-wt/MWI/OP1-326/1998/G1P[8]
RVA/Human-wt/MWI/MW1-800/1999/G1P[8]
RVA/Human-wt/MWI/BTY1Z6/2017/G1P[8]
RVA/Human-wt/MWI/BTY2B0/2019/G1P[8]
RVA/Human-wt/MWI/BTY16W/2015/G1P[8]
RVA/Human-wt/MWI/BTY16L/2015/G1P[8]
RVA/Human-wt/ITA/AV21/2010/G9P[8]
RVA/Human-wt/IDN/STM453/2018/G1P[8]
RVA/Human-wt/STM430/2017/G12P[8]
RVA/Human-wt/NGA/NGR Ref/2017/GXP[X]
RVA/Human-wt/JPN/RVA-U14/2015/G1P[8]
RVA/Human-wt/IND/CMC 00048/2013/G9P[8]
RVA/Human-wt/BGD/RV-MMC2019-1/2019/G3P[8]
RVA/Human-wt/BGD/RV-MMC2019-4/2019/G3P[8]
RVA/Human-wt/MWI/BTY2EM/2019/G3P[8]
RVA/Human-wt/MWI/CHX11U/2019/G3P[8]
RVA/Human-wt/MWI/CHX11S/2019/G3P[8]
RVA/Human-wt/MWI/BTY2BD/2018/G3P 8
RVA/Human-wt/MWI/BTY2BD/2018/G3P[8]
RVA/Human-wt/MWI/BTY2GA/2019/G3P[8]
0.05

**c. VP4**

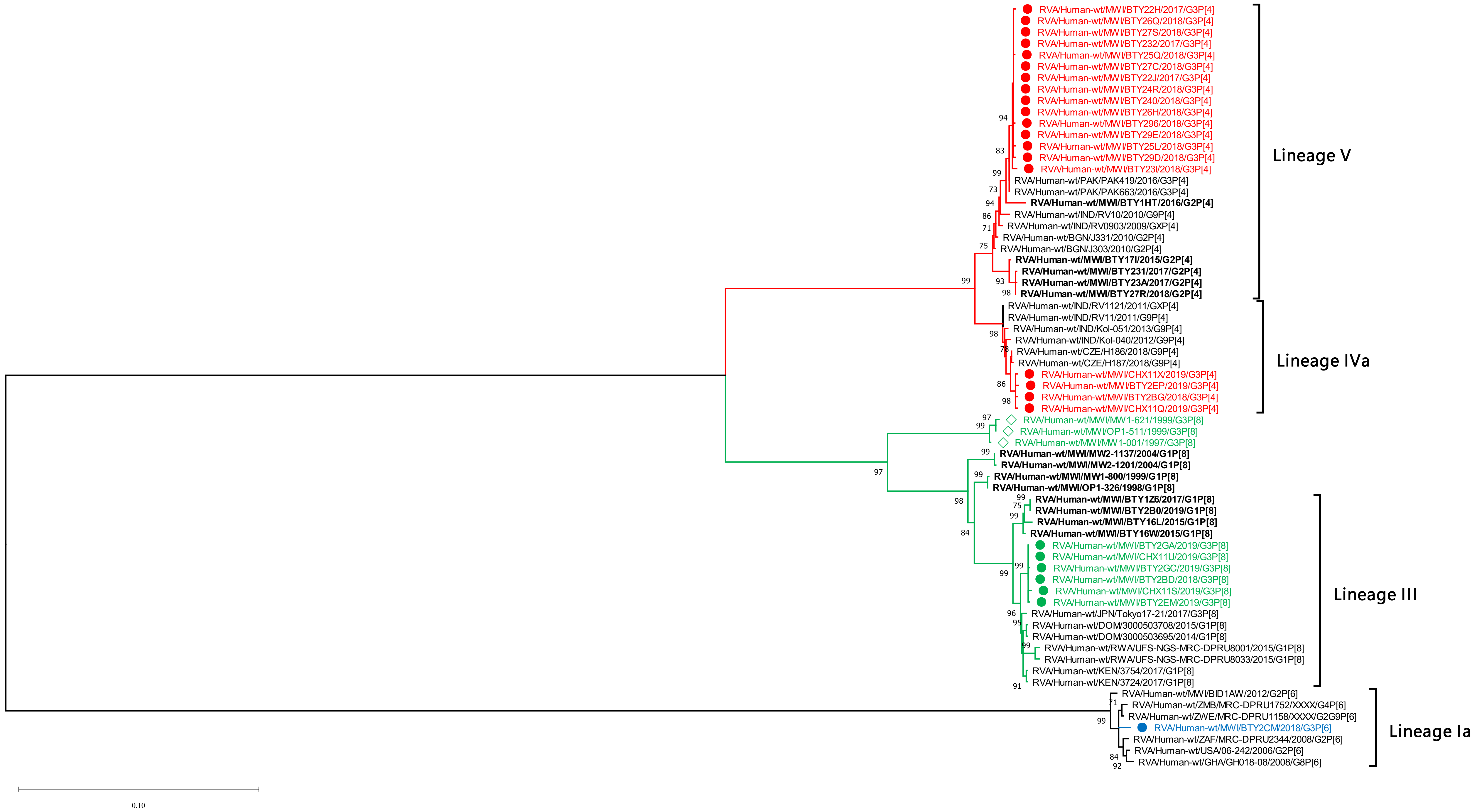

RVA/Human-wt/MWI/BTY22H/2017/G3P[4]
RVA/Human-wt/MWI/BTY26Q/2018/G3P[4]
RVA/Human-wt/MWI/BTY27S/2018/G3P[4]
RVA/Human-wt/MWI/BTY232/2017/G3P[4]
RVA/Human-wt/MWI/BTY25Q/2018/G3P[4]
RVA/Human-wt/MWI/BTY27C/2018/G3P[4]
RVA/Human-wt/MWI/BTY22J/2017/G3P[4]
RVA/Human-wt/MWI/BTY24R/2018/G3P[4]
RVA/Human-wt/MWI/BTY240/2018/G3P[4]
RVA/Human-wt/MWI/BTY26H/2018/G3P[4]
RVA/Human-wt/MWI/BTY296/2018/G3P[4]
RVA/Human-wt/MWI/BTY29E/2018/G3P[4]
RVA/Human-wt/MWI/BTY25L/2018/G3P[4]
RVA/Human-wt/MWI/BTY29D/2018/G3P[4]
RVA/Human-wt/MWI/BTY23I/2018/G3P[4]
RVA/Human-wt/PAK/PAK419/2016/G3P[4]
RVA/Human-wt/PAK/PAK663/2016/G3P[4]
RVA/Human-wt/MWI/BTY1HT/2016/G2P[4]
RVA/Human-wt/IND/RV10/2010/G9P[4]
RVA/Human-wt/IND/RV0903/2009/GXP[4]
RVA/Human-wt/BGN/J331/2010/G2P[4]
RVA/Human-wt/BGN/J303/2010/G2P[4]
RVA/Human-wt/MWI/BTY17I/2015/G2P[4]
RVA/Human-wt/MWI/BTY231/2017/G2P[4]
RVA/Human-wt/MWI/BTY23A/2017/G2P[4]
RVA/Human-wt/MWI/BTY27R/2018/G2P[4]
RVA/Human-wt/IND/RV1121/2011/GXP[4]
RVA/Human-wt/IND/RV11/2011/G9P[4]
RVA/Human-wt/IND/Kol-051/2013/G9P[4]
RVA/Human-wt/IND/Kol-040/2012/G9P[4]
RVA/Human-wt/CZE/H186/2018/G9P[4]
RVA/Human-wt/CZE/H187/2018/G9P[4]
RVA/Human-wt/MWI/CHX11X/2019/G3P[4]
RVA/Human-wt/MWI/BTY2EP/2019/G3P[4]
RVA/Human-wt/MWI/BTY2BG/2018/G3P[4]
RVA/Human-wt/MWI/CHX11Q/2019/G3P[4]
RVA/Human-wt/MWI/MW1-621/1999/G3P[8]
RVA/Human-wt/MWI/OP1-511/1999/G3P[8]
RVA/Human-wt/MWI/MW1-001/1997/G3P[8]
RVA/Human-wt/MWI/MW2-1137/2004/G1P[8]
RVA/Human-wt/MWI/MW2-1201/2004/G1P[8]
RVA/Human-wt/MWI/MW1-800/1999/G1P[8]
RVA/Human-wt/MWI/OP1-326/1998/G1P[8]
RVA/Human-wt/MWI/BTY1Z6/2017/G1P[8]
RVA/Human-wt/MWI/BTY2B0/2019/G1P[8]
RVA/Human-wt/MWI/BTY16L/2015/G1P[8]
RVA/Human-wt/MWI/BTY16W/2015/G1P[8]
RVA/Human-wt/MWI/BTY2GA/2019/G3P[8]
RVA/Human-wt/MWI/CHX11U/2019/G3P[8]
RVA/Human-wt/MWI/BTY2GC/2019/G3P[8]
RVA/Human-wt/MWI/BTY2BD/2018/G3P[8]
RVA/Human-wt/MWI/CHX11S/2019/G3P[8]
RVA/Human-wt/MWI/BTY2EM/2019/G3P[8]
RVA/Human-wt/JPN/Tokyo17-21/2017/G3P[8]
RVA/Human-wt/DOM/3000503708/2015/G1P[8]
RVA/Human-wt/DOM/3000503695/2014/G1P[8]
RVA/Human-wt/RWA/UFS-NGS-MRC-DPRU8001/2015/G1P[8]
RVA/Human-wt/RWA/UFS-NGS-MRC-DPRU8033/2015/G1P[8]
RVA/Human-wt/KEN/3754/2017/G1P[8]
RVA/Human-wt/KEN/3724/2017/G1P[8]
RVA/Human-wt/MWI/BID1AW/2012/G2P[6]
RVA/Human-wt/ZMB/MRC-DPRU1752/XXXX/G4P[6]
RVA/Human-wt/ZWE/MRC-DPRU1158/XXXX/G2G9P[6]
RVA/Human-wt/MWI/BTY2CM/2018/G3P[6]
RVA/Human-wt/ZAF/MRC-DPRU2344/2008/G2P[6]
RVA/Human-wt/USA/06-242/2006/G2P[6]
RVA/Human-wt/GHA/GH018-08/2008/G8P[6]
Lineage V
Lineage IVa
Lineage III
Lineage Ia
0.10

d. VP2

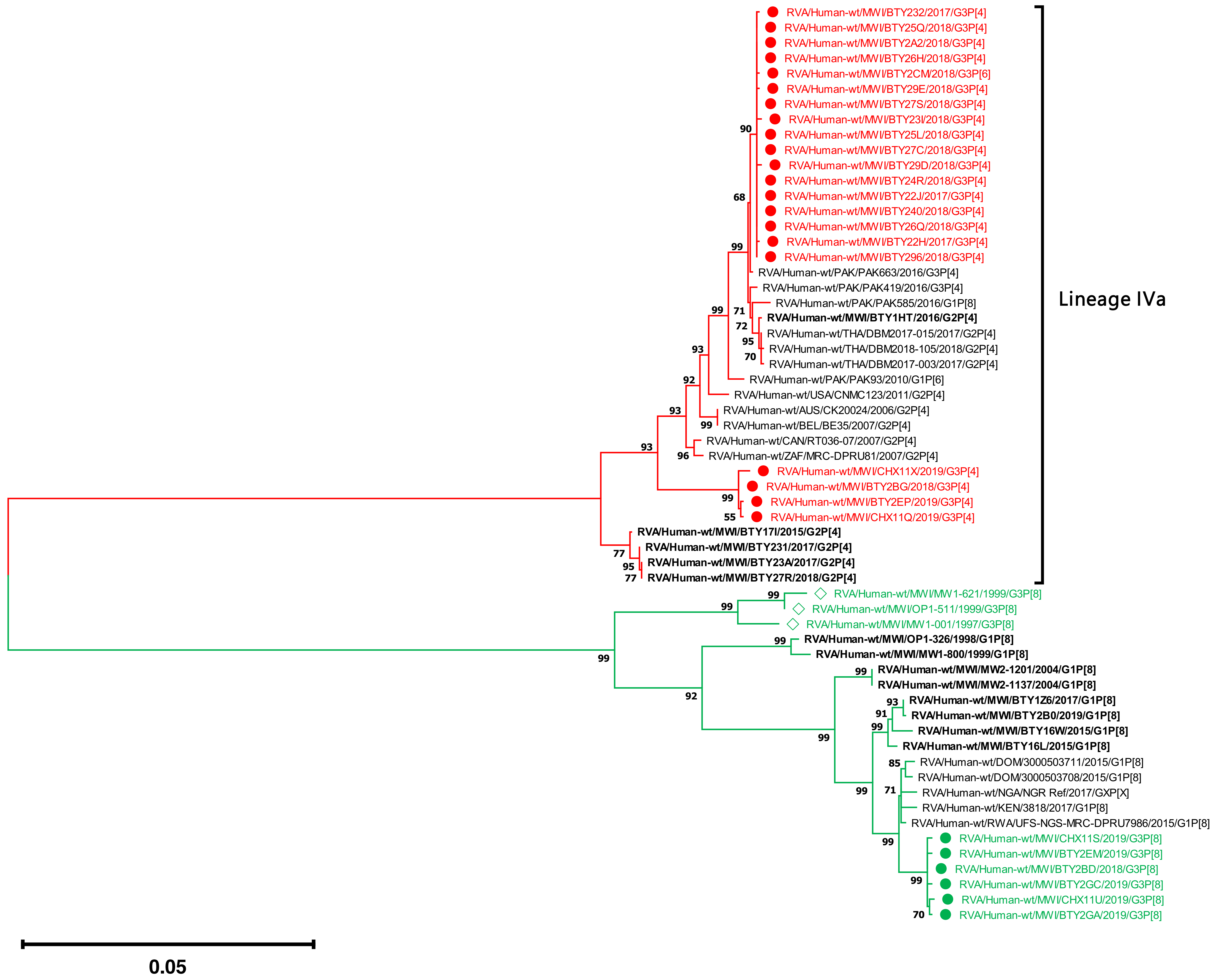
RVA/Human-wt/MWI/BTY232/2017/G3P[4]
RVA/Human-wt/MWI/BTY25Q/2018/G3P[4]
RVA/Human-wt/MWI/BTY2A2/2018/G3P[4]
RVA/Human-wt/MWI/BTY26H/2018/G3P[4]
RVA/Human-wt/MWI/BTY2CM/2018/G3P[6]
RVA/Human-wt/MWI/BTY29E/2018/G3P[4]
RVA/Human-wt/MWI/BTY27S/2018/G3P[4]
RVA/Human-wt/MWI/BTY23I/2018/G3P[4]
RVA/Human-wt/MWI/BTY25L/2018/G3P[4]
RVA/Human-wt/MWI/BTY27C/2018/G3P[4]
RVA/Human-wt/MWI/BTY29D/2018/G3P[4]
RVA/Human-wt/MWI/BTY24R/2018/G3P[4]
RVA/Human-wt/MWI/BTY22J/2017/G3P[4]
RVA/Human-wt/MWI/BTY240/2018/G3P[4]
RVA/Human-wt/MWI/BTY26Q/2018/G3P[4]
RVA/Human-wt/MWI/BTY22H/2017/G3P[4]
RVA/Human-wt/MWI/BTY296/2018/G3P[4]
RVA/Human-wt/PAK/PAK663/2016/G3P[4]
RVA/Human-wt/PAK/PAK419/2016/G3P[4]
RVA/Human-wt/PAK/PAK585/2016/G1P[8]
RVA/Human-wt/MWI/BTY1HT/2016/G2P[4]
RVA/Human-wt/THA/DBM2017-015/2017/G2P[4]
RVA/Human-wt/THA/DBM2018-105/2018/G2P[4]
RVA/Human-wt/THA/DBM2017-003/2017/G2P[4]
RVA/Human-wt/PAK/PAK93/2010/G1P[6]
RVA/Human-wt/USA/CNMC123/2011/G2P[4]
RVA/Human-wt/AUS/CK20024/2006/G2P[4]
RVA/Human-wt/BEL/BE35/2007/G2P[4]
RVA/Human-wt/CAN/RT036-07/2007/G2P[4]
RVA/Human-wt/ZAF/MRC-DPRU81/2007/G2P[4]
RVA/Human-wt/MWI/CHX11X/2019/G3P[4]
RVA/Human-wt/MWI/BTY2BG/2018/G3P[4]
RVA/Human-wt/MWI/BTY2EP/2019/G3P[4]
RVA/Human-wt/MWI/CHX11Q/2019/G3P[4]
RVA/Human-wt/MWI/BTY17I/2015/G2P[4]
RVA/Human-wt/MWI/BTY231/2017/G2P[4]
RVA/Human-wt/MWI/BTY23A/2017/G2P[4]
RVA/Human-wt/MWI/BTY27R/2018/G2P[4]
Lineage IVa
RVA/Human-wt/MWI/MW1-621/1999/G3P[8]
RVA/Human-wt/MWI/OP1-511/1999/G3P[8]
RVA/Human-wt/MWI/MW1-001/1997/G3P[8]
RVA/Human-wt/MWI/OP1-326/1998/G1P[8]
RVA/Human-wt/MWI/MW1-800/1999/G1P[8]
RVA/Human-wt/MWI/MW2-1201/2004/G1P[8]
RVA/Human-wt/MWI/MW2-1137/2004/G1P[8]
RVA/Human-wt/MWI/BTY1Z6/2017/G1P[8]
RVA/Human-wt/MWI/BTY2B0/2019/G1P[8]
RVA/Human-wt/MWI/BTY16W/2015/G1P[8]
RVA/Human-wt/MWI/BTY16L/2015/G1P[8]
RVA/Human-wt/DOM/3000503711/2015/G1P[8]
RVA/Human-wt/DOM/3000503708/2015/G1P[8]
RVA/Human-wt/NGA/NGR Ref/2017/GXP[X]
RVA/Human-wt/KEN/3818/2017/G1P[8]
RVA/Human-wt/RWA/UFS-NGS-MRC-DPRU7986/2015/G1P[8]
RVA/Human-wt/MWI/CHX11S/2019/G3P[8]
RVA/Human-wt/MWI/BTY2EM/2019/G3P[8]
RVA/Human-wt/MWI/BTY2BD/2018/G3P[8]
RVA/Human-wt/MWI/BTY2GC/2019/G3P[8]
RVA/Human-wt/MWI/CHX11U/2019/G3P[8]
RVA/Human-wt/MWI/BTY2GA/2019/G3P[8]
0.05

e. VP1

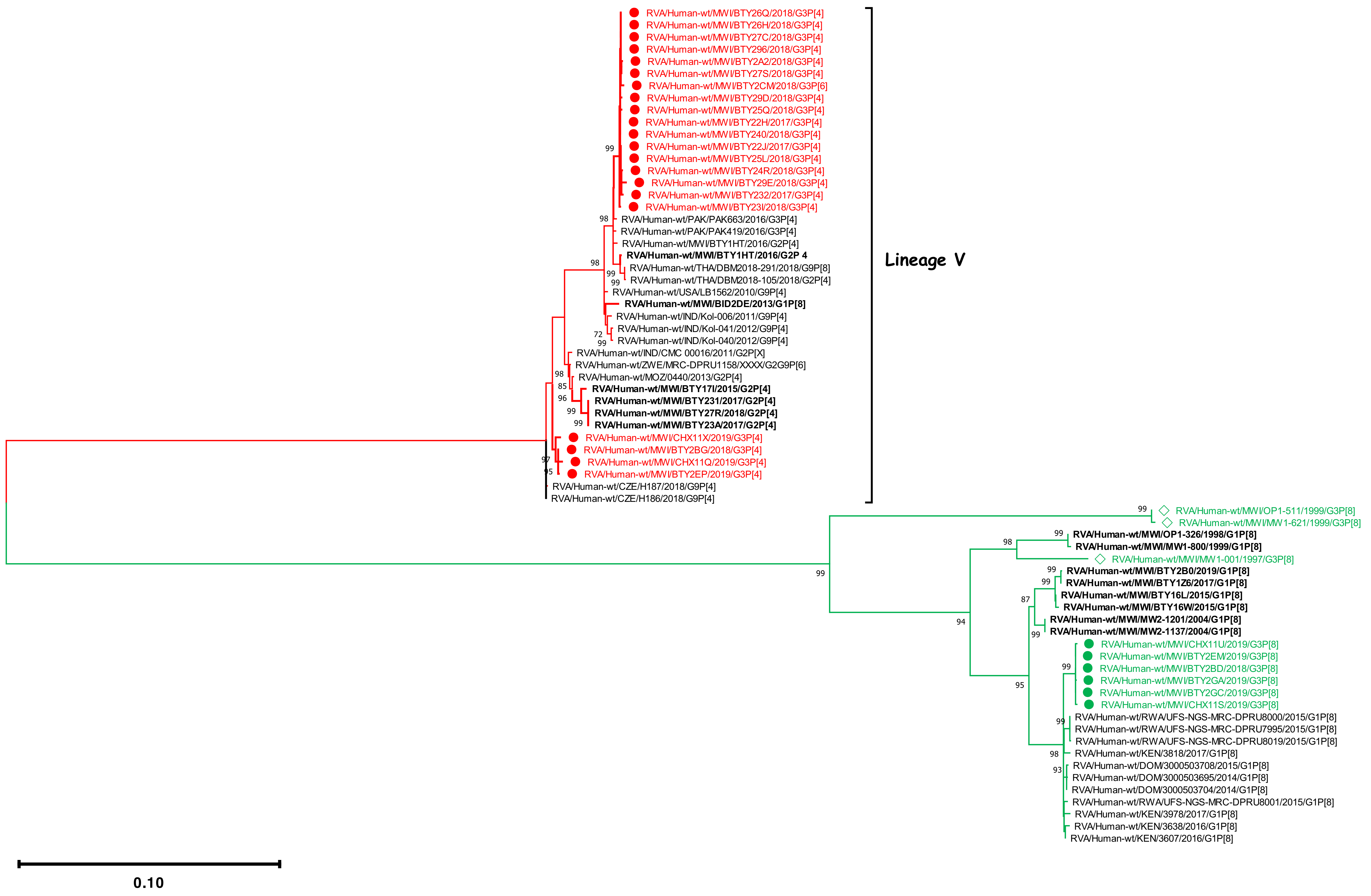

RVA/Human-wt/MWI/BTY26Q/2018/G3P[4]
RVA/Human-wt/MWI/BTY26H/2018/G3P[4]
RVA/Human-wt/MWI/BTY27C/2018/G3P[4]
RVA/Human-wt/MWI/BTY296/2018/G3P[4]
RVA/Human-wt/MWI/BTY2A2/2018/G3P[4]
RVA/Human-wt/MWI/BTY27S/2018/G3P[4]
RVA/Human-wt/MWI/BTY2CM/2018/G3P[6]
RVA/Human-wt/MWI/BTY29D/2018/G3P[4]
RVA/Human-wt/MWI/BTY25Q/2018/G3P[4]
RVA/Human-wt/MWI/BTY22H/2017/G3P[4]
RVA/Human-wt/MWI/BTY240/2018/G3P[4]
RVA/Human-wt/MWI/BTY22J/2017/G3P[4]
RVA/Human-wt/MWI/BTY25L/2018/G3P[4]
RVA/Human-wt/MWI/BTY24R/2018/G3P[4]
RVA/Human-wt/MWI/BTY29E/2018/G3P[4]
RVA/Human-wt/MWI/BTY232/2017/G3P[4]
RVA/Human-wt/MWI/BTY23I/2018/G3P[4]
RVA/Human-wt/PAK/PAK663/2016/G3P[4]
RVA/Human-wt/PAK/PAK419/2016/G3P[4]
RVA/Human-wt/MWI/BTY1HT/2016/G2P[4]
RVA/Human-wt/MWI/BTY1HT/2016/G2P 4
RVA/Human-wt/THA/DBM2018-291/2018/G9P[8]
RVA/Human-wt/THA/DBM2018-105/2018/G2P[4]
RVA/Human-wt/USA/LB1562/2010/G9P[4]
RVA/Human-wt/MWI/BID2DE/2013/G1P[8]
RVA/Human-wt/IND/Kol-006/2011/G9P[4]
RVA/Human-wt/IND/Kol-041/2012/G9P[4]
RVA/Human-wt/IND/Kol-040/2012/G9P[4]
RVA/Human-wt/IND/CMC 00016/2011/G2P[X]
RVA/Human-wt/ZWE/MRC-DPRU1158/XXXX/G2G9P[6]
RVA/Human-wt/MOZ/0440/2013/G2P[4]
RVA/Human-wt/MWI/BTY17I/2015/G2P[4]
RVA/Human-wt/MWI/BTY231/2017/G2P[4]
RVA/Human-wt/MWI/BTY27R/2018/G2P[4]
RVA/Human-wt/MWI/BTY23A/2017/G2P[4]
RVA/Human-wt/MWI/CHX11X/2019/G3P[4]
RVA/Human-wt/MWI/BTY2BG/2018/G3P[4]
RVA/Human-wt/MWI/CHX11Q/2019/G3P[4]
RVA/Human-wt/MWI/BTY2EP/2019/G3P[4]
RVA/Human-wt/CZE/H187/2018/G9P[4]
RVA/Human-wt/CZE/H186/2018/G9P[4]
Lineage V
RVA/Human-wt/MWI/OP1-511/1999/G3P[8]
RVA/Human-wt/MWI/MW1-621/1999/G3P[8]
RVA/Human-wt/MWI/OP1-326/1998/G1P[8]
RVA/Human-wt/MWI/MW1-800/1999/G1P[8]
RVA/Human-wt/MWI/MW1-001/1997/G3P[8]
RVA/Human-wt/MWI/BTY2B0/2019/G1P[8]
RVA/Human-wt/MWI/BTY1Z6/2017/G1P[8]
RVA/Human-wt/MWI/BTY16L/2015/G1P[8]
RVA/Human-wt/MWI/BTY16W/2015/G1P[8]
RVA/Human-wt/MWI/MW2-1201/2004/G1P[8]
RVA/Human-wt/MWI/MW2-1137/2004/G1P[8]
RVA/Human-wt/MWI/CHX11U/2019/G3P[8]
RVA/Human-wt/MWI/BTY2EM/2019/G3P[8]
RVA/Human-wt/MWI/BTY2BD/2018/G3P[8]
RVA/Human-wt/MWI/BTY2GA/2019/G3P[8]
RVA/Human-wt/MWI/BTY2GC/2019/G3P[8]
RVA/Human-wt/MWI/CHX11S/2019/G3P[8]
RVA/Human-wt/RWA/UFS-NGS-MRC-DPRU8000/2015/G1P[8]
RVA/Human-wt/RWA/UFS-NGS-MRC-DPRU7995/2015/G1P[8]
RVA/Human-wt/RWA/UFS-NGS-MRC-DPRU8019/2015/G1P[8]
RVA/Human-wt/KEN/3818/2017/G1P[8]
RVA/Human-wt/DOM/3000503708/2015/G1P[8]
RVA/Human-wt/DOM/3000503695/2014/G1P[8]
RVA/Human-wt/DOM/3000503704/2014/G1P[8]
RVA/Human-wt/RWA/UFS-NGS-MRC-DPRU8001/2015/G1P[8]
RVA/Human-wt/KEN/3978/2017/G1P[8]
RVA/Human-wt/KEN/3638/2016/G1P[8]
RVA/Human-wt/KEN/3607/2016/G1P[8]
0.10

f. NSP1

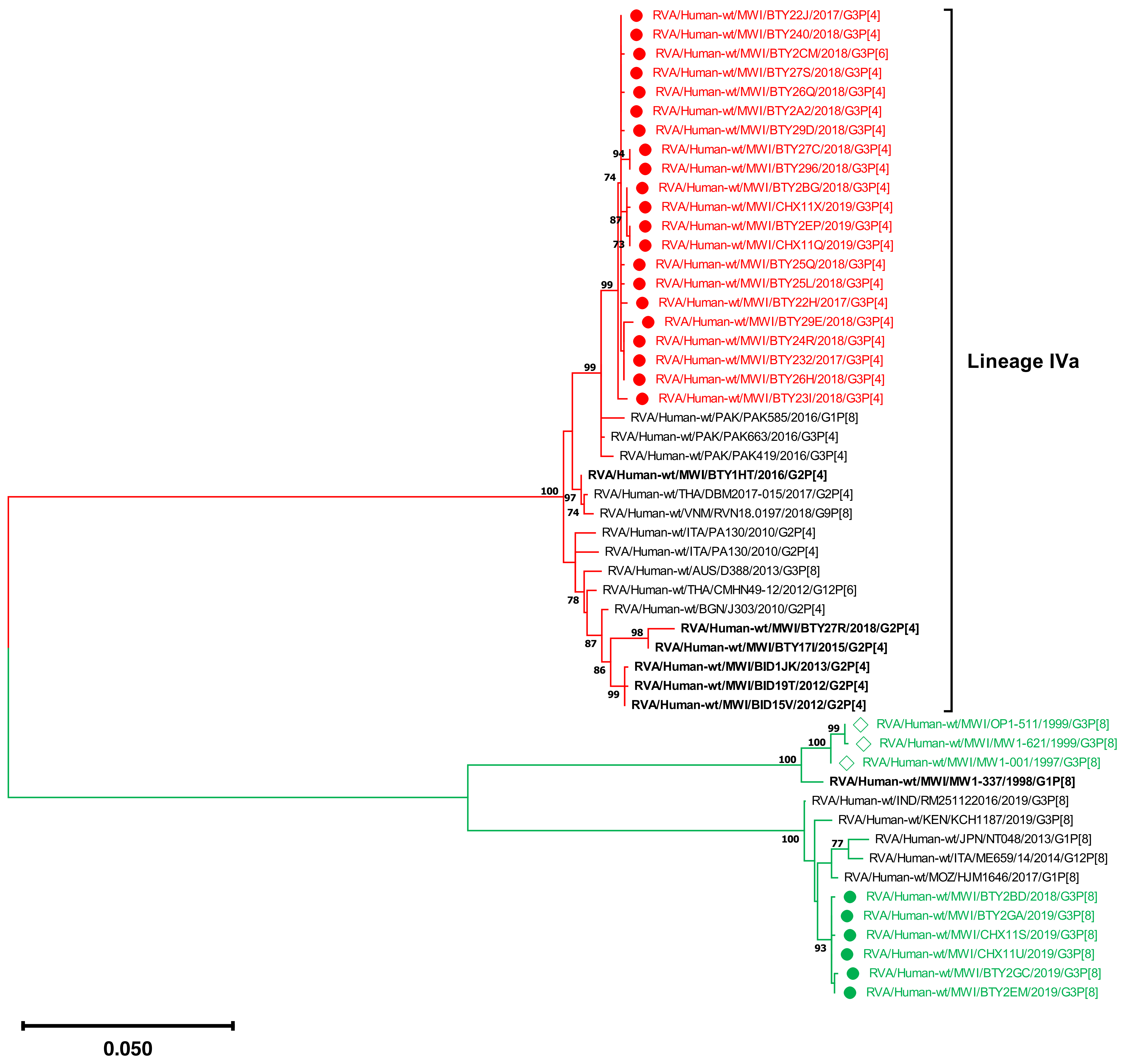
RVA/Human-wt/MWI/BTY22J/2017/G3P[4]
RVA/Human-wt/MWI/BTY240/2018/G3P[4]
RVA/Human-wt/MWI/BTY2CM/2018/G3P[6]
RVA/Human-wt/MWI/BTY27S/2018/G3P[4]
RVA/Human-wt/MWI/BTY26Q/2018/G3P[4]
RVA/Human-wt/MWI/BTY2A2/2018/G3P[4]
RVA/Human-wt/MWI/BTY29D/2018/G3P[4]
RVA/Human-wt/MWI/BTY27C/2018/G3P[4]
RVA/Human-wt/MWI/BTY296/2018/G3P[4]
RVA/Human-wt/MWI/BTY2BG/2018/G3P[4]
RVA/Human-wt/MWI/CHX11X/2019/G3P[4]
RVA/Human-wt/MWI/BTY2EP/2019/G3P[4]
RVA/Human-wt/MWI/CHX11Q/2019/G3P[4]
RVA/Human-wt/MWI/BTY25Q/2018/G3P[4]
RVA/Human-wt/MWI/BTY25L/2018/G3P[4]
RVA/Human-wt/MWI/BTY22H/2017/G3P[4]
RVA/Human-wt/MWI/BTY29E/2018/G3P[4]
RVA/Human-wt/MWI/BTY24R/2018/G3P[4]
RVA/Human-wt/MWI/BTY232/2017/G3P[4]
RVA/Human-wt/MWI/BTY26H/2018/G3P[4]
RVA/Human-wt/MWI/BTY23I/2018/G3P[4]
RVA/Human-wt/PAK/PAK585/2016/G1P[8]
RVA/Human-wt/PAK/PAK663/2016/G3P[4]
RVA/Human-wt/PAK/PAK419/2016/G3P[4]
RVA/Human-wt/MWI/BTY1HT/2016/G2P[4]
RVA/Human-wt/THA/DBM2017-015/2017/G2P[4]
RVA/Human-wt/VNM/RVN18.0197/2018/G9P[8]
RVA/Human-wt/ITA/PA130/2010/G2P[4]
RVA/Human-wt/ITA/PA130/2010/G2P[4]
RVA/Human-wt/AUS/D388/2013/G3P[8]
RVA/Human-wt/THA/CMHN49-12/2012/G12P[6]
RVA/Human-wt/BGN/J303/2010/G2P[4]
RVA/Human-wt/MWI/BTY27R/2018/G2P[4]
RVA/Human-wt/MWI/BTY17I/2015/G2P[4]
RVA/Human-wt/MWI/BID1JK/2013/G2P[4]
RVA/Human-wt/MWI/BID19T/2012/G2P[4]
RVA/Human-wt/MWI/BID15V/2012/G2P[4]
Lineage IVa
RVA/Human-wt/MWI/OP1-511/1999/G3P[8]
RVA/Human-wt/MWI/MW1-621/1999/G3P[8]
RVA/Human-wt/MWI/MW1-001/1997/G3P[8]
RVA/Human-wt/MWI/MW1-337/1998/G1P[8]
RVA/Human-wt/IND/RM251122016/2019/G3P[8]
RVA/Human-wt/KEN/KCH1187/2019/G3P[8]
RVA/Human-wt/JPN/NT048/2013/G1P[8]
RVA/Human-wt/ITA/ME659/14/2014/G12P[8]
RVA/Human-wt/MOZ/HJM1646/2017/G1P[8]
RVA/Human-wt/MWI/BTY2BD/2018/G3P[8]
RVA/Human-wt/MWI/BTY2GA/2019/G3P[8]
RVA/Human-wt/MWI/CHX11S/2019/G3P[8]
RVA/Human-wt/MWI/CHX11U/2019/G3P[8]
RVA/Human-wt/MWI/BTY2GC/2019/G3P[8]
RVA/Human-wt/MWI/BTY2EM/2019/G3P[8]
0.050

**g. NSP3**

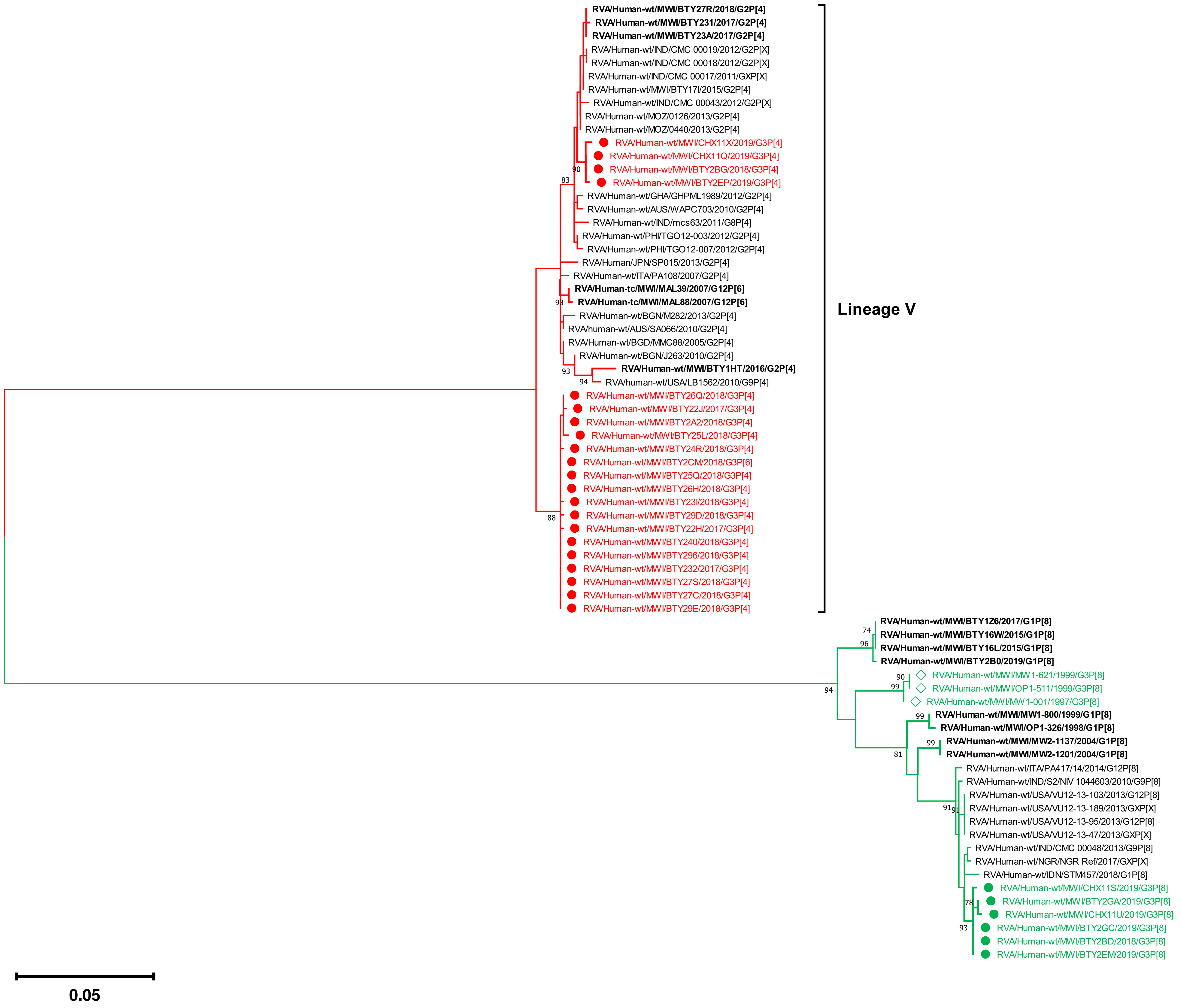

RVA/Human-wt/MWI/BTY27R/2018/G2P[4]
RVA/Human-wt/MWI/BTY231/2017/G2P[4]
RVA/Human-wt/MWI/BTY23A/2017/G2P[4]
RVA/Human-wt/IND/CMC 00019/2012/G2P[X]
RVA/Human-wt/IND/CMC 00018/2012/G2P[X]
RVA/Human-wt/IND/CMC 00017/2011/GXP[X]
RVA/Human-wt/MWI/BTY17I/2015/G2P[4]
RVA/Human-wt/IND/CMC 00043/2012/G2P[X]
RVA/Human-wt/MOZ/0126/2013/G2P[4]
RVA/Human-wt/MOZ/0440/2013/G2P[4]
RVA/Human-wt/MWI/CHX11X/2019/G3P[4]
RVA/Human-wt/MWI/CHX11Q/2019/G3P[4]
RVA/Human-wt/MWI/BTY2BG/2018/G3P[4]
RVA/Human-wt/MWI/BTY2EP/2019/G3P[4]
RVA/Human-wt/GHA/GHPML1989/2012/G2P[4]
RVA/Human-wt/AUS/WAPC703/2010/G2P[4]
RVA/Human-wt/IND/mcs63/2011/G8P[4]
RVA/Human-wt/PHI/TGO12-003/2012/G2P[4]
RVA/Human-wt/PHI/TGO12-007/2012/G2P[4]
RVA/Human/JPN/SP015/2013/G2P[4]
RVA/Human-wt/ITA/PA108/2007/G2P[4]
RVA/Human-tc/MWI/MAL39/2007/G12P[6]
RVA/Human-tc/MWI/MAL88/2007/G12P[6]
RVA/Human-wt/BGN/M282/2013/G2P[4]
RVA/human-wt/AUS/SA066/2010/G2P[4]
RVA/Human-wt/BGD/MMC88/2005/G2P[4]
RVA/Human-wt/BGN/J263/2010/G2P[4]
RVA/Human-wt/MWI/BTY1HT/2016/G2P[4]
RVA/human-wt/USA/LB1562/2010/G9P[4]
RVA/Human-wt/MWI/BTY26Q/2018/G3P[4]
RVA/Human-wt/MWI/BTY22J/2017/G3P[4]
RVA/Human-wt/MWI/BTY2A2/2018/G3P[4]
RVA/Human-wt/MWI/BTY25L/2018/G3P[4]
RVA/Human-wt/MWI/BTY24R/2018/G3P[4]
RVA/Human-wt/MWI/BTY2CM/2018/G3P[6]
RVA/Human-wt/MWI/BTY25Q/2018/G3P[4]
RVA/Human-wt/MWI/BTY26H/2018/G3P[4]
RVA/Human-wt/MWI/BTY23I/2018/G3P[4]
RVA/Human-wt/MWI/BTY29D/2018/G3P[4]
RVA/Human-wt/MWI/BTY22H/2017/G3P[4]
RVA/Human-wt/MWI/BTY240/2018/G3P[4]
RVA/Human-wt/MWI/BTY296/2018/G3P[4]
RVA/Human-wt/MWI/BTY232/2017/G3P[4]
RVA/Human-wt/MWI/BTY27S/2018/G3P[4]
RVA/Human-wt/MWI/BTY27C/2018/G3P[4]
RVA/Human-wt/MWI/BTY29E/2018/G3P[4]
Lineage V
RVA/Human-wt/MWI/BTY1Z6/2017/G1P[8]
RVA/Human-wt/MWI/BTY16W/2015/G1P[8]
RVA/Human-wt/MWI/BTY16L/2015/G1P[8]
RVA/Human-wt/MWI/BTY2B0/2019/G1P[8]
RVA/Human-wt/MWI/MW1-621/1999/G3P[8]
RVA/Human-wt/MWI/OP1-511/1999/G3P[8]
RVA/Human-wt/MWI/MW1-001/1997/G3P[8]
RVA/Human-wt/MWI/MW1-800/1999/G1P[8]
RVA/Human-wt/MWI/OP1-326/1998/G1P[8]
RVA/Human-wt/MWI/MW2-1137/2004/G1P[8]
RVA/Human-wt/MWI/MW2-1201/2004/G1P[8]
RVA/Human-wt/ITA/PA417/14/2014/G12P[8]
RVA/Human-wt/IND/S2/NIV 1044603/2010/G9P[8]
RVA/Human-wt/USA/VU12-13-103/2013/G12P[8]
RVA/Human-wt/USA/VU12-13-189/2013/GXP[X]
RVA/Human-wt/USA/VU12-13-95/2013/G12P[8]
RVA/Human-wt/USA/VU12-13-47/2013/GXP[X]
RVA/Human-wt/IND/CMC 00048/2013/G9P[8]
RVA/Human-wt/NGR/NGR Ref/2017/GXP[X]
RVA/Human-wt/IDN/STM457/2018/G1P[8]
RVA/Human-wt/MWI/CHX11S/2019/G3P[8]
RVA/Human-wt/MWI/BTY2GA/2019/G3P[8]
RVA/Human-wt/MWI/CHX11U/2019/G3P[8]
RVA/Human-wt/MWI/BTY2GC/2019/G3P[8]
RVA/Human-wt/MWI/BTY2BD/2018/G3P[8]
RVA/Human-wt/MWI/BTY2EM/2019/G3P[8]
0.05

h. NSP4

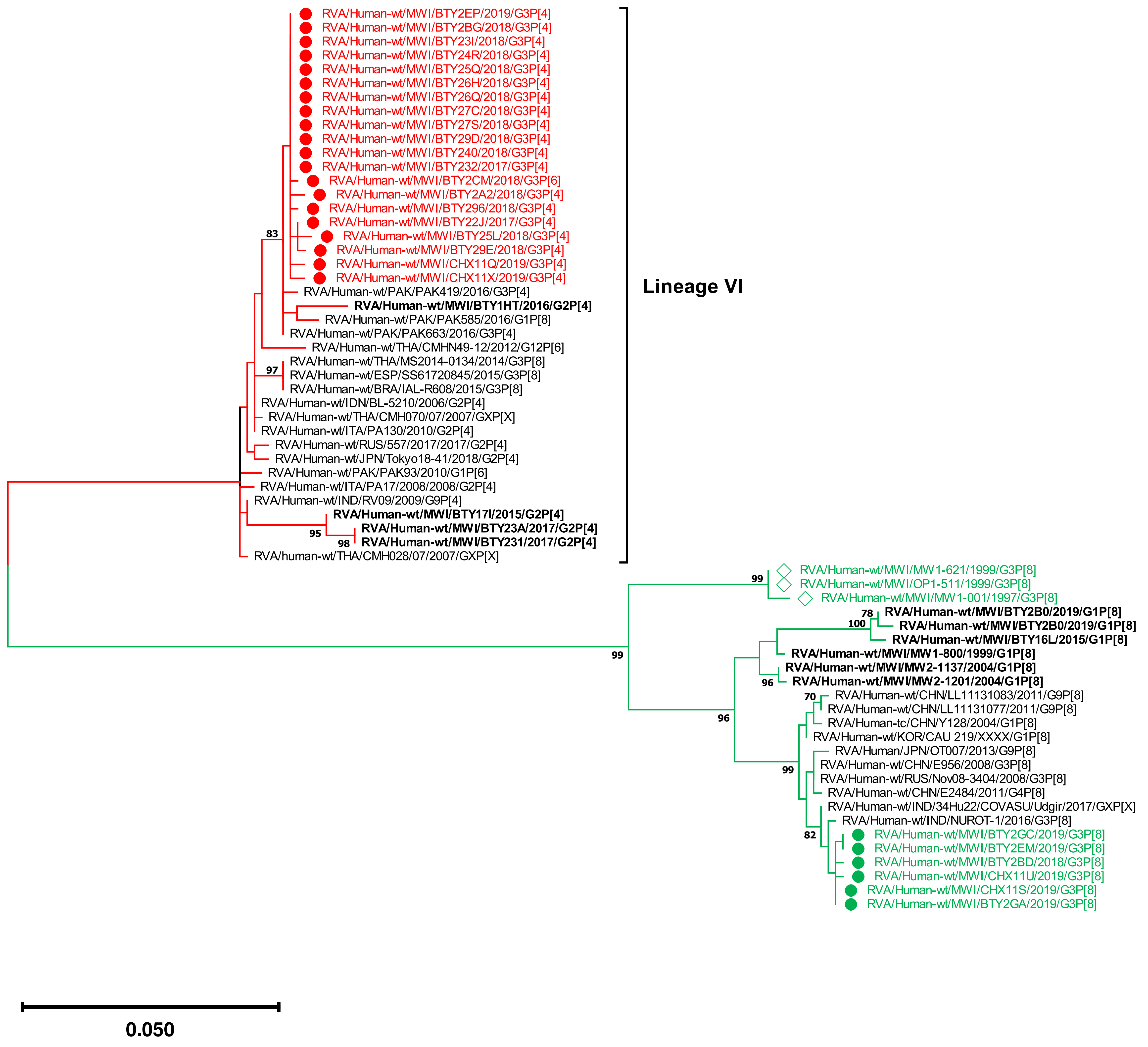
RVA/Human-wt/MWI/BTY2EP/2019/G3P[4]
RVA/Human-wt/MWI/BTY2BG/2018/G3P[4]
RVA/Human-wt/MWI/BTY23I/2018/G3P[4]
RVA/Human-wt/MWI/BTY24R/2018/G3P[4]
RVA/Human-wt/MWI/BTY25Q/2018/G3P[4]
RVA/Human-wt/MWI/BTY26H/2018/G3P[4]
RVA/Human-wt/MWI/BTY26Q/2018/G3P[4]
RVA/Human-wt/MWI/BTY27C/2018/G3P[4]
RVA/Human-wt/MWI/BTY27S/2018/G3P[4]
RVA/Human-wt/MWI/BTY29D/2018/G3P[4]
RVA/Human-wt/MWI/BTY240/2018/G3P[4]
RVA/Human-wt/MWI/BTY232/2017/G3P[4]
RVA/Human-wt/MWI/BTY2CM/2018/G3P[6]
RVA/Human-wt/MWI/BTY2A2/2018/G3P[4]
RVA/Human-wt/MWI/BTY296/2018/G3P[4]
RVA/Human-wt/MWI/BTY22J/2017/G3P[4]
RVA/Human-wt/MWI/BTY25L/2018/G3P[4]
RVA/Human-wt/MWI/BTY29E/2018/G3P[4]
RVA/Human-wt/MWI/CHX11Q/2019/G3P[4]
RVA/Human-wt/MWI/CHX11X/2019/G3P[4]
83
RVA/Human-wt/PAK/PAK419/2016/G3P[4]
RVA/Human-wt/MWI/BTY1HT/2016/G2P[4]
RVA/Human-wt/PAK/PAK585/2016/G1P[8]
RVA/Human-wt/PAK/PAK663/2016/G3P[4]
RVA/Human-wt/THA/CMHN49-12/2012/G12P[6]
RVA/Human-wt/THA/MS2014-0134/2014/G3P[8]
97
RVA/Human-wt/ESP/SS61720845/2015/G3P[8]
RVA/Human-wt/BRA/IAL-R608/2015/G3P[8]
RVA/Human-wt/IDN/BL-5210/2006/G2P[4]
RVA/Human-wt/THA/CMH070/07/2007/GXP[X]
RVA/Human-wt/ITA/PA130/2010/G2P[4]
RVA/Human-wt/RUS/557/2017/2017/G2P[4]
RVA/Human-wt/JPN/Tokyo18-41/2018/G2P[4]
RVA/Human-wt/PAK/PAK93/2010/G1P[6]
RVA/Human-wt/ITA/PA17/2008/2008/G2P[4]
RVA/Human-wt/IND/RV09/2009/G9P[4]
RVA/Human-wt/MWI/BTY17I/2015/G2P[4]
95
RVA/Human-wt/MWI/BTY23A/2017/G2P[4]
98
RVA/Human-wt/MWI/BTY231/2017/G2P[4]
RVA/human-wt/THA/CMH028/07/2007/GXP[X]
Lineage VI
99
RVA/Human-wt/MWI/MW1-621/1999/G3P[8]
RVA/Human-wt/MWI/OP1-511/1999/G3P[8]
RVA/Human-wt/MWI/MW1-001/1997/G3P[8]
78
RVA/Human-wt/MWI/BTY2B0/2019/G1P[8]
100
RVA/Human-wt/MWI/BTY2B0/2019/G1P[8]
RVA/Human-wt/MWI/BTY16L/2015/G1P[8]
99
RVA/Human-wt/MWI/MW1-800/1999/G1P[8]
RVA/Human-wt/MWI/MW2-1137/2004/G1P[8]
96
RVA/Human-wt/MWI/MW2-1201/2004/G1P[8]
70
RVA/Human-wt/CHN/LL11131083/2011/G9P[8]
RVA/Human-wt/CHN/LL11131077/2011/G9P[8]
96
RVA/Human-tc/CHN/Y128/2004/G1P[8]
RVA/Human-wt/KOR/CAU 219/XXXX/G1P[8]
RVA/Human/JPN/OT007/2013/G9P[8]
99
RVA/Human-wt/CHN/E956/2008/G3P[8]
RVA/Human-wt/RUS/Nov08-3404/2008/G3P[8]
RVA/Human-wt/CHN/E2484/2011/G4P[8]
RVA/Human-wt/IND/34Hu22/COVASU/Udgir/2017/GXP[X]
RVA/Human-wt/IND/NUROT-1/2016/G3P[8]
82
RVA/Human-wt/MWI/BTY2GC/2019/G3P[8]
RVA/Human-wt/MWI/BTY2EM/2019/G3P[8]
RVA/Human-wt/MWI/BTY2BD/2018/G3P[8]
RVA/Human-wt/MWI/CHX11U/2019/G3P[8]
RVA/Human-wt/MWI/CHX11S/2019/G3P[8]
RVA/Human-wt/MWI/BTY2GA/2019/G3P[8]
0.050

**i. NSP5**

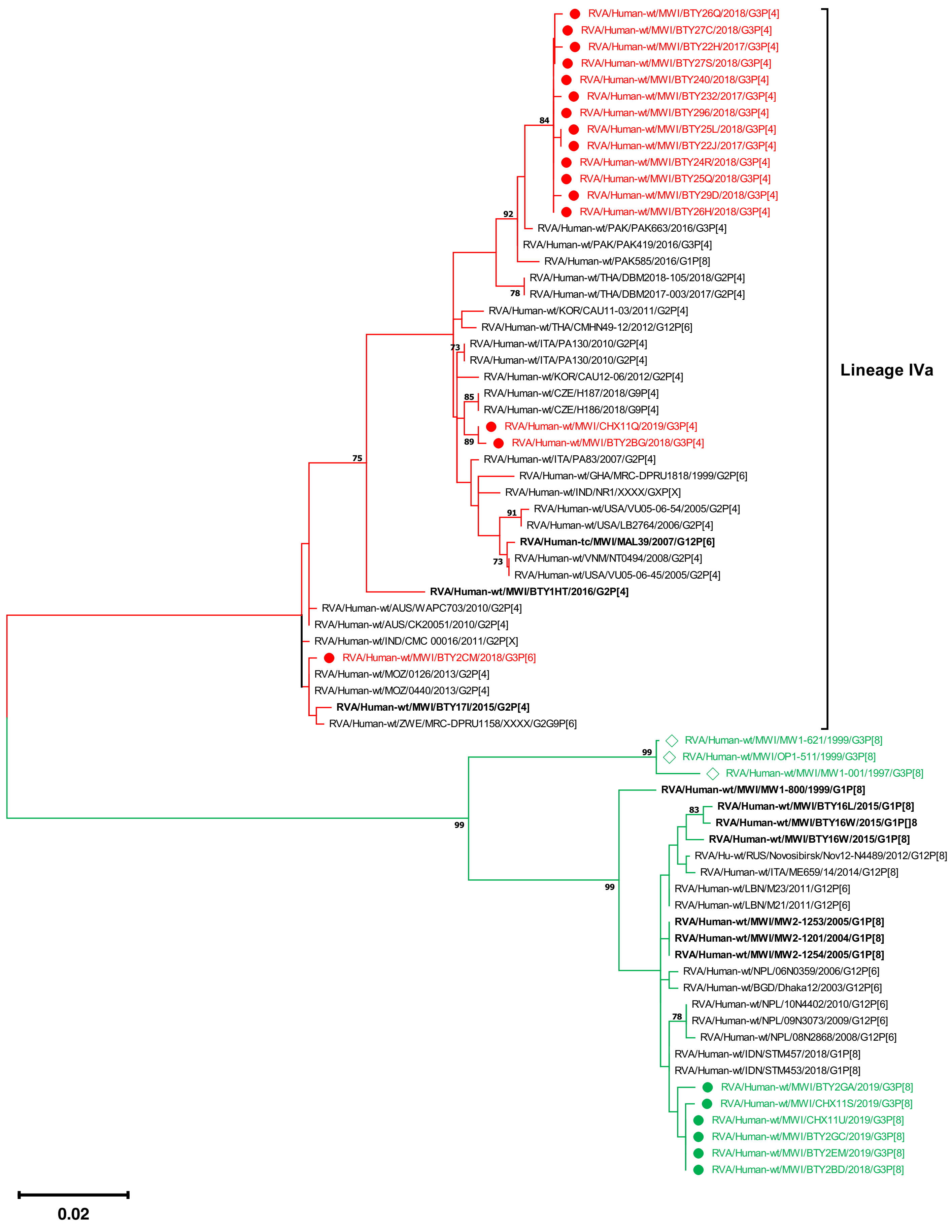
RVA/Human-wt/MWI/BTY26Q/2018/G3P[4]
RVA/Human-wt/MWI/BTY27C/2018/G3P[4]
RVA/Human-wt/MWI/BTY22H/2017/G3P[4]
RVA/Human-wt/MWI/BTY27S/2018/G3P[4]
RVA/Human-wt/MWI/BTY240/2018/G3P[4]
RVA/Human-wt/MWI/BTY232/2017/G3P[4]
RVA/Human-wt/MWI/BTY296/2018/G3P[4]
RVA/Human-wt/MWI/BTY25L/2018/G3P[4]
RVA/Human-wt/MWI/BTY22J/2017/G3P[4]
RVA/Human-wt/MWI/BTY24R/2018/G3P[4]
RVA/Human-wt/MWI/BTY25Q/2018/G3P[4]
RVA/Human-wt/MWI/BTY29D/2018/G3P[4]
RVA/Human-wt/MWI/BTY26H/2018/G3P[4]
84
RVA/Human-wt/PAK/PAK663/2016/G3P[4]
RVA/Human-wt/PAK/PAK419/2016/G3P[4]
92
RVA/Human-wt/PAK585/2016/G1P[8]
RVA/Human-wt/THA/DBM2018-105/2018/G2P[4]
78
RVA/Human-wt/THA/DBM2017-003/2017/G2P[4]
RVA/Human-wt/KOR/CAU11-03/2011/G2P[4]
RVA/Human-wt/THA/CMHN49-12/2012/G12P[6]
73
RVA/Human-wt/ITA/PA130/2010/G2P[4]
RVA/Human-wt/ITA/PA130/2010/G2P[4]
RVA/Human-wt/KOR/CAU12-06/2012/G2P[4]
85
RVA/Human-wt/CZE/H187/2018/G9P[4]
RVA/Human-wt/CZE/H186/2018/G9P[4]
RVA/Human-wt/MWI/CHX11Q/2019/G3P[4]
89
RVA/Human-wt/MWI/BTY2BG/2018/G3P[4]
RVA/Human-wt/ITA/PA83/2007/G2P[4]
RVA/Human-wt/GHA/MRC-DPRU1818/1999/G2P[6]
RVA/Human-wt/IND/NR1/XXXX/GXP[X]
91
RVA/Human-wt/USA/VU05-06-54/2005/G2P[4]
RVA/Human-wt/USA/LB2764/2006/G2P[4]
RVA/Human-tc/MWI/MAL39/2007/G12P[6]
73
RVA/Human-wt/VNM/NT0494/2008/G2P[4]
RVA/Human-wt/USA/VU05-06-45/2005/G2P[4]
75
RVA/Human-wt/MWI/BTY1HT/2016/G2P[4]
RVA/Human-wt/AUS/WAPC703/2010/G2P[4]
RVA/Human-wt/AUS/CK20051/2010/G2P[4]
RVA/Human-wt/IND/CMC 00016/2011/G2P[X]
RVA/Human-wt/MWI/BTY2CM/2018/G3P[6]
RVA/Human-wt/MOZ/0126/2013/G2P[4]
RVA/Human-wt/MOZ/0440/2013/G2P[4]
RVA/Human-wt/MWI/BTY17I/2015/G2P[4]
RVA/Human-wt/ZWE/MRC-DPRU1158/XXXX/G2G9P[6]
Lineage IVa
99
RVA/Human-wt/MWI/MW1-621/1999/G3P[8]
RVA/Human-wt/MWI/OP1-511/1999/G3P[8]
RVA/Human-wt/MWI/MW1-001/1997/G3P[8]
99
RVA/Human-wt/MWI/MW1-800/1999/G1P[8]
83
RVA/Human-wt/MWI/BTY16L/2015/G1P[8]
RVA/Human-wt/MWI/BTY16W/2015/G1P[]8
RVA/Human-wt/MWI/BTY16W/2015/G1P[8]
RVA/Hu-wt/RUS/Novosibirsk/Nov12-N4489/2012/G12P[8]
RVA/Human-wt/ITA/ME659/14/2014/G12P[8]
99
RVA/Human-wt/LBN/M23/2011/G12P[6]
RVA/Human-wt/LBN/M21/2011/G12P[6]
RVA/Human-wt/MWI/MW2-1253/2005/G1P[8]
RVA/Human-wt/MWI/MW2-1201/2004/G1P[8]
RVA/Human-wt/MWI/MW2-1254/2005/G1P[8]
RVA/Human-wt/NPL/06N0359/2006/G12P[6]
RVA/Human-wt/BGD/Dhaka12/2003/G12P[6]
RVA/Human-wt/NPL/10N4402/2010/G12P[6]
78
RVA/Human-wt/NPL/09N3073/2009/G12P[6]
RVA/Human-wt/NPL/08N2868/2008/G12P[6]
RVA/Human-wt/IDN/STM457/2018/G1P[8]
RVA/Human-wt/IDN/STM453/2018/G1P[8]
RVA/Human-wt/MWI/BTY2GA/2019/G3P[8]
RVA/Human-wt/MWI/CHX11S/2019/G3P[8]
RVA/Human-wt/MWI/CHX11U/2019/G3P[8]
RVA/Human-wt/MWI/BTY2GC/2019/G3P[8]
RVA/Human-wt/MWI/BTY2EM/2019/G3P[8]
RVA/Human-wt/MWI/BTY2BD/2018/G3P[8]
0.02

**Supplementary Figure S3**. **Maximum likelihood (ML) phylogenetic trees of Malawian G3 strains with global reference strains sharing a high nucleotide sequence similarity to Malawian strains**. Only strains with a complete open reading frame were included in the analysis. The GTR evolutionary model with Gamma heterogeneity across nucleotide sites was used for phylogenetic inference. Bootstrap values ≥70% are shown adjacent to each branch node. Malawian Wa-like and DS-1-like G3 strains are denoted by green and red colours respectively. Circles represent post-vaccine strains while diamonds represent pre-vaccine strains (A-H). Shows ML trees for VP7, VP4, VP6, VP1-VP2, NSP1, NSP3-NSP5 with a global lineage definition system for VP7, VP4 and DS-1-like genome segments while Wa-like genome segments have global as well as local reference strains

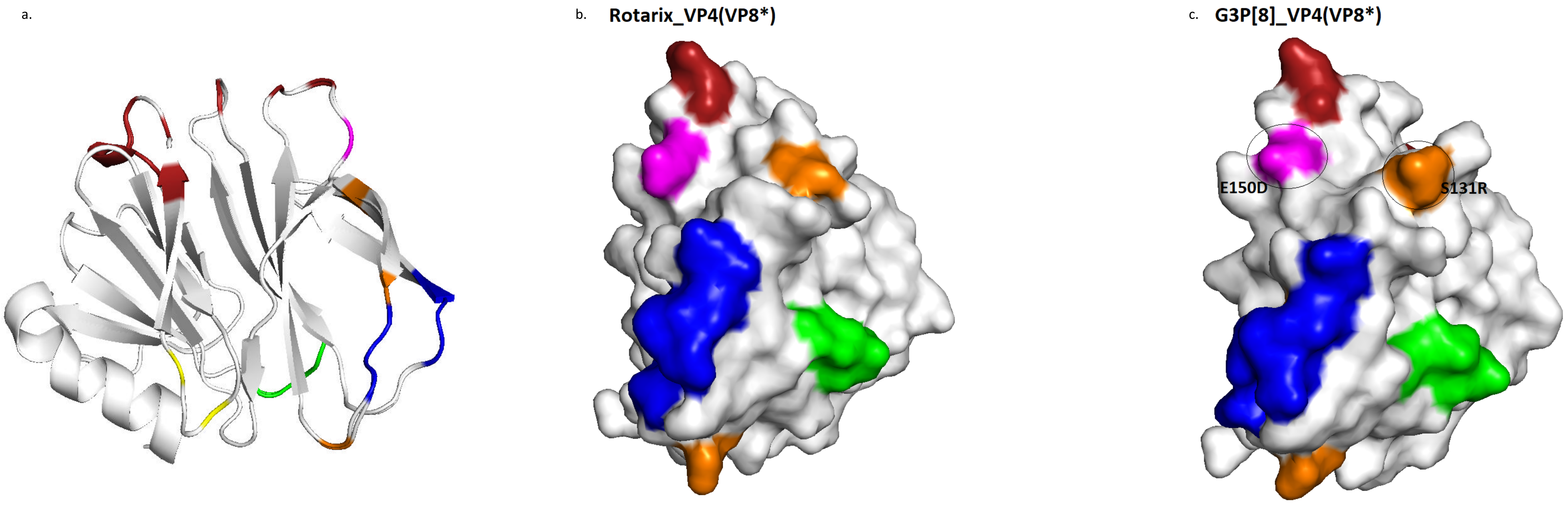


**Supplementary Figure S4. Protein model structures comparing the antigenic regions within VP8* of the VP4 protein between Rotarix and the G3P[8] strains**. (a) Perfect alignment of superimposed VP4 structures exhibiting few differences between RV1 and P[8] genotypes associated with G3 rotaviruses in Malawi. VP8*-1 in red, VP8*-2 in blue, VP8*-3 in green and VP8*-4 in yellow. Amino acid position 150 and 131 are colored in magenta and orange respectively. (b and c) surface structural differences between Rotarix and P[8] genotypes associated with rotaviruses in Malawi.
